# Two Novel Aspochalasins from the Gut Fungus *Aspergillus* sp. Z4

**DOI:** 10.3390/md16100343

**Published:** 2018-09-20

**Authors:** Xinyang Li, Wanjing Ding, Pinmei Wang, Jinzhong Xu

**Affiliations:** Institute of Marine Biology, Ocean College, Zhejiang University, Zhoushan 316021, China; lxinyang2014@126.com (X.L.); wading@zju.edu.cn (W.D.); wangpinmei@zju.edu.cn (P.W.)

**Keywords:** aspochalasin, tricyclic fused, gut fungus, cytotoxicity

## Abstract

Two novel aspochalasins, tricochalasin A (**1**) and aspochalasin A2 (**2**), along with three known compounds (**3**–**5**) have been isolated from the different culture broth of *Aspergillus* sp., which was found in the gut of a marine isopod *Ligia oceanica*. Compound **1** contains a rare 5/6/6 tricyclic ring fused with the aspochalasin skeleton. The structures were determined on the basis of electrospray ionisation mass spectroscopy (ESIMS), nuclear magnetic resonance (NMR) spectral data, and the absolute configurations were further confirmed by modified Mosher’s method. Cytotoxicity against the prostate cancer PC3 cell line were assayed by the MTT method. Compound **3** showed strong activity while the remaining compounds showed weak activity.

## 1. Introduction

**Figure 1:**
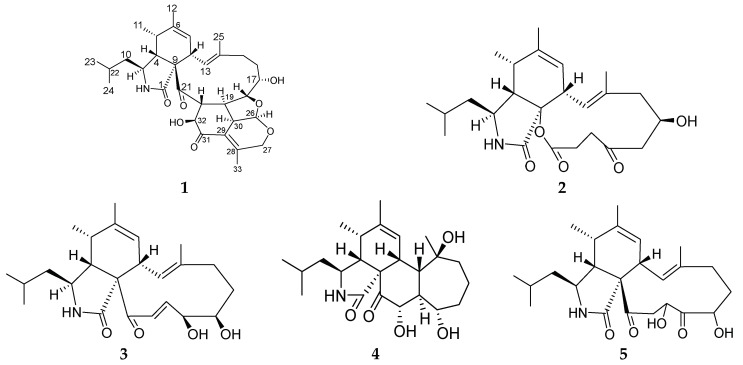
Structures of compounds **1**–**5**.

Aspochalasins constitute a subgroup within the small group of cytochalasans, which are fungal secondary metabolites known for varieties of biological activities [1]. These include cytotoxic [2,3,4], anti human immunodeficiency virus (HIV) [5], immunomodulatory [6], and nematicidal activity [7]. So far, more than 200 cytochalasan analogues have been reported [1]. Structurally, this group of compounds contains one isoindole unit fused with one macrocyclic ring. Isotope-labeling experiments have revealed that cytochalasans originate from an acetyl- and methionine-derived polyketide chain and the attachment of an amino acid precursors such as Leu, Phe, Ala and Trp [8,9]. With diverse oxygenated regions in the macrocyclic ring, there are several unusual analogues among these known compounds including chaetochalasin A [10], aspergillin PZ [11], spicochalasin A [12], epicochalasines A and B [13]. In our ongoing search for new bioactive metabolites of marine fungi, some new compounds have been purified from the marine-derived fungus Z4 [14,15,16,17], and one strain isolated from the gut of the marine isopod *Ligia oceanica*. In order to find more novel natural products from this fungus, we employed the OSMAC (one strain, many compounds) approach by varying the culture conditions of Z4. Two new cytochalasans, aspochalasins **1** and **2**, in addition to three known cytochalasans, aspochalasins **3**–**5** (Figure 1) were purified when cultured in media 2216E and rice. Herein we present the isolation, structure elucidation, and cytotoxic activity of these aspochalasins.

## 2. Results and Discussion

Compound **1** was purified as a colorless solid. The molecular formula C_32_H_43_NO_7_ with 12 degrees of unsaturation was established by the positive mode quasi-molecular ion peaks at *m*/*z* 554.3107 for [M + H]^+^ (calcd. 554.3073 for C_32_H_44_NO_7_) and *m*/*z* 576.2930 for [M + Na]^+^ (calcd. 576.2893 for C_32_H_43_NO_7_Na) combined with 1D nuclear magnetic resonance (NMR) data. The ^1^H NMR spectrum recorded in CDCl_3_ together with heteronuclear single quantum coherence (HSQC) spectra revealed six methyl groups [*δ*_H_ 0.93 (3H, d, *J* = 6.4 Hz), 0.96 (3H, d, *J* = 6.4 Hz), 1.18 (3H, d, *J* = 7.1 Hz), 1.57 (3H, s), 1.77 (3H, s), 1.85 (3H, s)], two olefinic protons [*δ*_H_ 5.45 (1H, br s), *δ*_H_ 5.70 (1H, d, *J* = 11.4 Hz)], three oxygenated methine hydrogen atoms [*δ*_H_ 4.35 (1H, d, *J* = 6.29 Hz), *δ*_H_ 4.42 (1H, dd, *J* = 3.6, 11.1 Hz), *δ*_H_ 3.86 (1H, d, *J* = 11.3 Hz)], one oxygenated methylene proton [*δ*_H_ 4.79 (2H, m)], one proton of acetal group [*δ*_H_ 5.91 (1H, d, *J* = 3.9 Hz)] and other 18 aliphatic protons. The ^13^C NMR and distortionless enhancement by polarization transfer (DEPT) spectra of **1** indicated 32 carbon resonances ascribed to two ketone carbonyl, one amide carbonyl, six olefinic carbons, five oxygenated carbons including one acetal carbon, 12 aliphatic carbon atoms and six methyl carbons (Table 1). These features characteristically revealed the structure of **1** to be an aspochalasin skeleton. The excess number of carbon atoms when compared to the previously isolated and reported aspochalasin derivatives [18,19], indicated that **1** was an unusual one.

**Figure 2:**
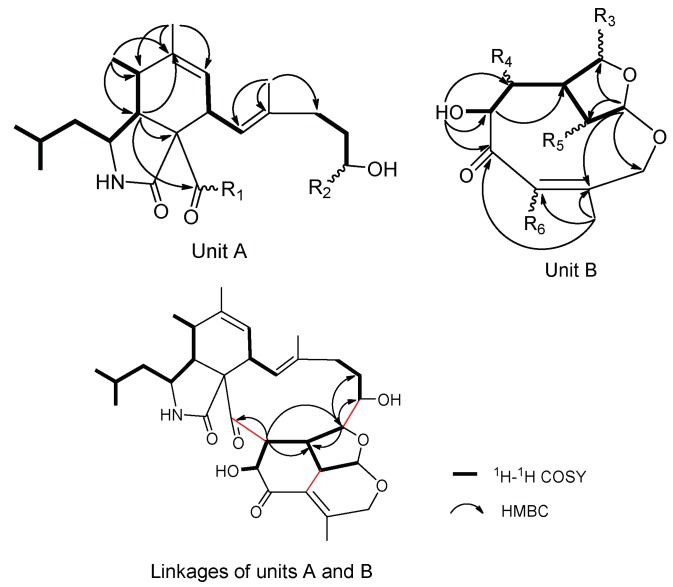
Partial structures of **1** based on ^1^H-^1^H correlation spectroscopy (COSY) and heteronuclear multiple bond correlation (HMBC) spectra.

The planar structure of **1** was elucidated by 2D NMR spectrum. The ^1^H-^1^H correlation spectroscopy (COSY) cross peaks (Figure S8 in Supplementary Materials) of Me-23 (*δ*_H_ 0.93)/Me-24 (*δ*_H_ 0.96)/H-22 (*δ*_H_ 1.53)/H-10 (*δ*_H_ 1.49, 1.25)/H-3 (*δ*_H_ 2.97)/H-4 (*δ*_H_ 2.81)/H-5 (*δ*_H_ 2.67)/Me-11 (*δ*_H_ 1.18), H-7 (*δ*_H_ 5.45)/H-8 (*δ*_H_ 3.61)/H-13 (*δ*_H_ 5.70), H-15 (*δ*_H_ 2.07)/H-16 (*δ*_H_ 1.50, 1.30)/H-17 (*δ*_H_ 4.35) and heteronuclear multiple bond correlation (HMBC) correlations (Figure S9) from Me-12 (*δ*_H_ 1.77) to C-5 (*δ*_C_ 35.9), C-6 (*δ*_C_ 140.6) and C-7 (*δ*_C_ 125.6), from Me-25 (*δ*_H_ 1.57) to C-13 (*δ*_C_ 125.1), C-14 (*δ*_C_ 135.9) and C-15 (*δ*_C_ 36.9) established unit A, possessing a (2-methylpropyl) isoindolone moiety, which had two positions vacant to be linked, R1 and R2 (Figure 2). 11 carbon signals remained. The ^1^H-^1^H COSY cross peaks of H-26 (*δ*_H_ 5.91)/H-30 (*δ*_H_ 3.59)/H-19 (*δ*_H_ 2.25)/H-18 (*δ*_H_ 3.86) and H-19/H-20 (*δ*_H_ 3.51)/H-32 (*δ*_H_ 4.42)/OH-32 (*δ*_H_ 4.58), together with HMBC interactions from Me-33 (*δ*_H_ 1.85) to C-29 (*δ*_C_ 127.6), C-28 (*δ*_C_ 158.1) and C-31 (*δ*_C_ 197.0), from H-20 and OH-32 to C-31 were preliminarily attributed to unit B (Figure 2). Furthermore, the HMBC correlations from H-19 and H-20 to C-21 (*δ*_C_ 214.2), from H-18 to C-16 (*δ*_C_ 29.3), C-17 (*δ*_C_ 68.6) and C-19 connected R1 with R4, and R2 with R3 respectively. To satisfy the unsaturation, R5 and R6 were linked. Thus, the gross planar structure of **1** was established.

**Figure 3:**
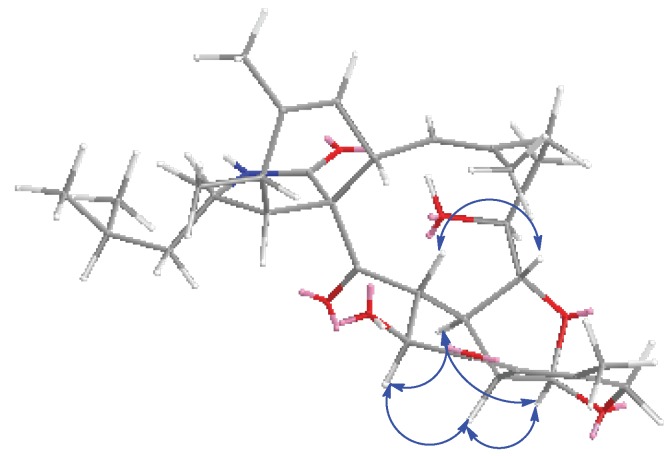
Key nuclear Overhauser effect spectroscopy (NOESY) correlations of **1**.

**Figure 4:**
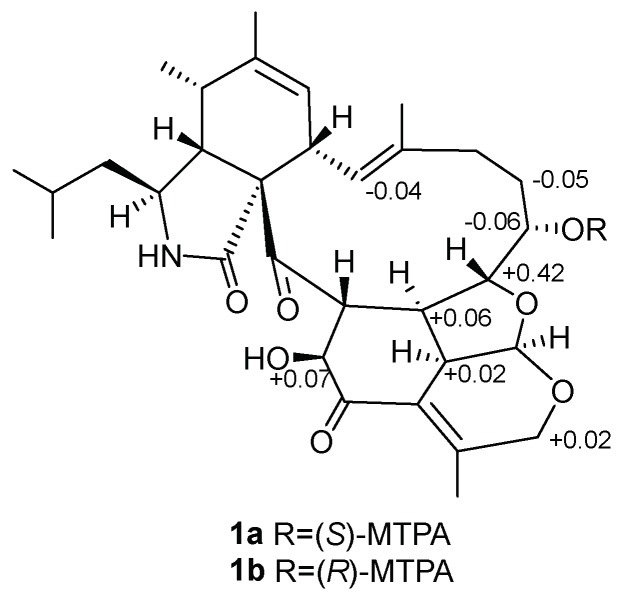
Δ*δ* values (in ppm) = *δ_S_*-*δ_R_* obtained for (*S*)- and (*R*)-α-methoxy-α-(trifluoromethyl) phenylacetyl (MPTA)-Cl esters **1a** and **1b**.

The relative stereochemistry of **1** was determined with the help of ^1^H NMR coupling constants, nuclear Overhauser effect spectroscopy (NOESY) experiments, and comparison with those of reported aspochalasins. The NOESY cross peaks (Figure S10) among H-5, H-4 and H-8 demonstrated the relative configurations of the isoindolone moiety in accordance with those of reported cytochalasans [20,21]. For the macrocyclic part, the NOESY correlations of H-17, H-18 and H-20 suggested they were cofacial. The double peak of H-18 with big coupling constants between H-19 reflected the *trans*-orientation of these two atoms. For the penta-heterocycle moiety, both H-19 and H-30 were located at the joint of three cycles, which elucidated the axial bond in these two atoms. Additionally, the NOESY correlations between H-19, H-30, H-26 and H-32 established that these moieties had the same orientation (Figure 3). The modified Mosher’s method using (*S*)/(*R*)-α-methoxy-α-(trifluoromethyl) phenylacetyl (MPTA)-Cl was applied to assign the absolute configuration of **1**. The positive and negative value disposition (Δ*δ^S-R^*) of the Mosher’s ester derivatives (**1a** and **1b**) established the absolute configuration of C-17 as *S* (Figure 4). It is noteworthy that in all natural cytochalasans, so far, the stereochemistry of perhydroisoindol-1-one moiety is the same [4,21] which assigned the absolute configurations for C-3, C-4, C-5,C-8 and C-9 as 3*S*, 4*R*, 5*S*, 8*S*, 9*S*, respectively. Therefore, the complete absolute stereochemistry of **1** could be assigned as 3*S*, 4*R*, 5*S*, 8*S*, 9*S*, 17*S*, 18*R*, 19*R*, 20*R*, 26*R*, 30*S*, 32*S* and named as Tricochalasin A.

**Figure 5:**
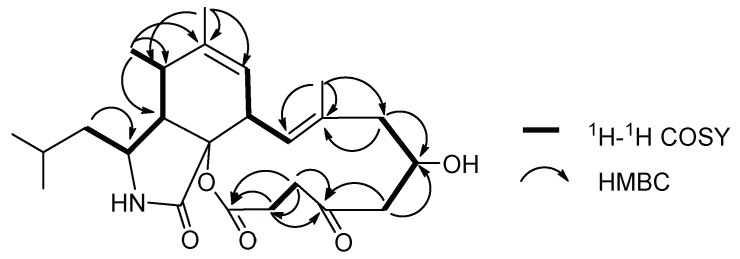
^1^H-^1^H COSY and key HMBC correlations of **2**.

Compound **2** was isolated as a white solid. Its molecular formula was deduced to be C_24_H_35_NO_5_ (8 degrees of unsaturation) by positive-mode high-resolution electrospray ionization mass spectrometry (HR-ESIMS) ion peaks at *m*/*z* 418.2589 [M + H]^+^ (calcd. 418.2549 for C_24_H_36_NO_5_) and *m*/*z* 440.2406 [M + Na]^+^ (calcd. 440.2368 for C_24_H_35_NO_5_Na), combined with 1D NMR data. Analysis of the ^1^H NMR and ^13^C NMR spectrum data recorded in CDCl_3_ revealed the presence of (2-methylpropyl) isoindolone moiety similar to those found in **1**. ^13^C NMR and DEPT spectra showed 24 carbon resonances including one ketone carbonyl (*δ*_C_ 208.9), one ester/lactone carbonyl (*δ*_C_ 171.9), one amide carbonyl (*δ*_C_ 172.4), four olefinic carbon (*δ*_C_ 123.5, 123.8, 138.2 and 140.9), five aliphatic methylene carbons (*δ*_C_ 46.8, 33.1, 32.3, 29.0 and 34.1), six aliphatic methine carbons (*δ*_C_ 25.6, 52.3, 52.4, 34.8, 41.1 and 75.1), five methyl signals (*δ*_C_ 21.1, 23.8, 14.3, 20.1 and 17.7) and one quaternary carbon (*δ*_C_ 86.5) (Table 1). These features characteristically suggested **2** belongs to the same structural family as **1**. Comparison with reported aspochalasin derivatives indicated that **2** was similar to aspochalasin A1 [22]. The planar structure of **2** was elucidated by ^1^H-^1^H COSY and ^1^H-^13^C HMBC experiments (Figure 5). ^1^H-^1^H COSY cross peaks (Figure S20) of the H-15 (*δ*_H_ 2.14, 2.27)/H-16 (*δ*_H_ 4.42)/H-17 (*δ*_H_ 2.04, 2.28) established the position of the hydroxyl group at C-16 (*δ*_C_ 75.1) and the H-19 (*δ*_H_ 2.32, 3.02)/H-20 (*δ*_H_ 2.44, 2.62) pairs located the ketone carbonyl group at C-18 (*δ*_C_ 208.9). The key HMBC correlations (Figure S21) from H-17 to C-16 and C-18, from H-15 to C-16 and C-14 (*δ*_C_ 138.2) and from H-19, H-20 to C-18 and C-21 (*δ*_C_ 171.9) also demonstrated the structural features.

**Figure 6:**
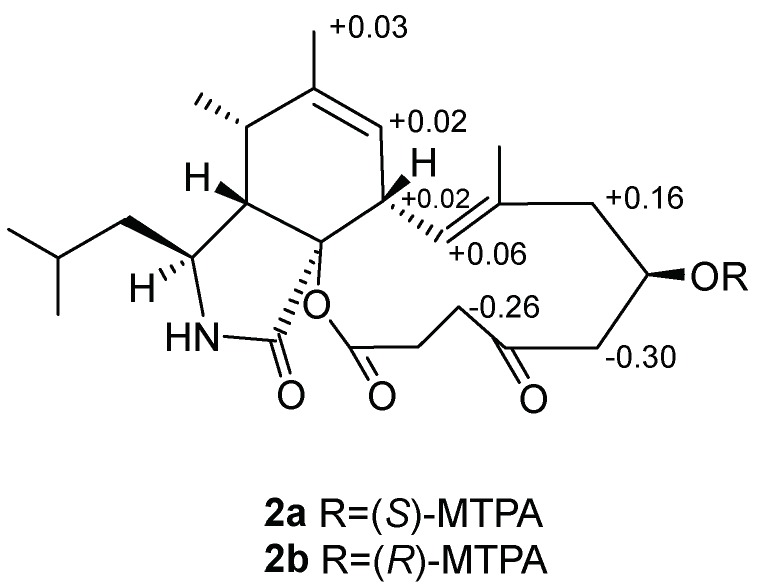
Δ*δ* values (in ppm) = *δ_S_*-*δ_R_* obtained for (*S*)- and (*R*)-MPTA esters **2a** and **2b**.

The absolute configuration of C-16 in **2** was established by the convenient Mosher’s ester. The difference in chemical shift values of the easters **2a** and **2b** was calculated to assign the absolute configuration at C-16 as *R* (Figure 6). Thus, the absolute configuration of **2** was deduced as 3*S*, 4*R*, 5*S*, 8*S*, 9*S*, 16*R* and named as aspochalasin A2.

NMR data of compounds **3**–**5** was in full agreement with the previously reported values for aspochalasins D (**3**) [23], aspergilluchalasin (**4**) [24] and aspochalasins T (**5**) [25].

All compounds were tested for their in vitro cytotoxicity against the prostate cancer PC3 cell lines by the MTT method, using doxorubicin (ADR) as positive control. As shown in Table 2, compound **3** showed strong activity against PC3 cell line, while others showed weak activities against it.

## 3. Materials and Methods 

### 3.1. General Experimental Procedures

Optical rotations were measured on a Jasco P-1010 polarimeter. The ultraviolet (UV) absorption spectra were measured in MeOH on a METASH UV-8000 spectrophotometer. The infrared (IR) spectra were recorded using a Bruker Vector 22 spectrometer (film) and an Avatar 370 Fourier-transform–infrared (FT–IR) spectrometer (KBr disk). NMR spectra were recorded in Chloroform-*d* (ALDRICH, St. Louis, MO, USA) with tetramethylsilane as an internal standard or Pyridine-*d*_5_ (ALDRICH, St. Louis, MO, USA), using a Bruker AVANCE-III 500 MHz NMR spectrometer (Brucker, Ettlingen, Germany). HR-ESIMS data were obtained on an Agilent 6224 TOF LC/MS (Agilent, Santa Clara, CA, USA) (positive mode; infusion rate: 0.6 mL/min; capillary voltages: 4000 V; nebulizer: 50 psig; drying gas: 10mL/min; gas temperature: 325 °C).

Column chromatography (CC) was performed with silica (100–200 and 200–300 mesh, Qingdao Haiyang Chemical Co., Ltd., Qingdao, China).

### 3.2. Fungal Material

The fungal colony (Z4) was isolated from marine isopod *Ligia oceanica* which was collected in seaside of Dinghai in Zhoushan, Zhejiang Province of China in December 2011. The fungal Z4 was determined as *Aspergillus* sp. by 18S rDNA analysis and preserved in China Center for Type Culture Collection (CCTCC No.M2013631).

### 3.3. Fermentation, Extraction, and Isolation of Strain Z4

Two different media were used: A—500 mL Erlenmeyer-flasks (400 × 200 mL, a total of 80 L) each containing 200 mL of 2216E liquid media (QingDao Hopebio-Technology Co., Ltd., Qingdao, China) and B—40 500 mL Erlenmeyer-flasks each containing 40 g of rice with 45 mL 3% artificial seawater. Both medium were incubated at 28 °C for 30 days in static condition.

Medium A was filtered and extracted with equivoluminal EtOAc for 3 times to yield 5 g of extract, which was fractioned by silica gel column chromatography (CC) eluted in gradient from petroleum ether-EtOAc (100:1–2:1) to CH_2_Cl_2_-MeOH (50:1–0:100). 8 fractions were collected based on thin layer chromatography (TLC) analysis. Fr. 5 (2.5 g) was fractionated by CC over silica gel using a gradient of CH_2_Cl_2_-MeOH (100:1–0:100) as a mobile phase to provide eight subfractions (Frs. 5-1 to 5-8). Fr. 5-4 (0.5 g) was further purified by CC over silica gel using a gradient of cyclohexane(C)-E(3:1–1:1) to yield nine subfractions (Frs. 5-4-1 to 5-4-9). Fr. 5-4-5 (90 mg) were purified by semi-preparative octadecyl-silica high-performance liquid chromatography (ODS-HPLC) (COSMOSIL PACKED COLUMN, 5C18-MS-II column, 10ID × 250 mm, 3 mL/min, 54% acetonitrile in H_2_O) to obtain compound **2** (3 mg). Fr. 5-4-9 (37 mg) were purified by semi-preparative ODS-HPLC (3 mL/min, 52% MeOH in H_2_O) to obtain compound **5** (17 mg). Each flask of B medium was extracted 3 times with EtOAc (60 mL) yielding 39 g of extract. CC over silica gel using a gradient of CH_2_Cl_2_-MeOH (100:1–0:100) as the eluents to afforded seven fractions based on TLC analysis. Fr. 3 (900 mg) was then purified by CC over silica gel with a CH_2_Cl_2_-MeOH gradient from 100:1–10:1 to afford 10 subfractions (Frs. 3-1 to 3-10), among which, Fr. 3-6 (28 mg) was further purified by semi-preparative ODS-HPLC (4 mL/min, 75% MeOH in H_2_O) to obtain compounds **1** (3 mg). Fr. 6 (4 g) was purified by CC over silica gel with a CH_2_Cl_2_-MeOH gradient from 50:1–5:1 to afford six subfractions (Frs. 6-1 to 6-6). Fr. 6-2 (76 mg) was purified by semi-preparative ODS-HPLC (4 mL/min, 75% MeOH in H_2_O) to obtain compounds **4** (13 mg). Fr. 6-3 (28 mg) was purified by semi-preparative ODS-HPLC (4 mL/min, 80% MeOH in H_2_O) to yield compound **3** (1 mg).

Tricochalasin A (**1**): colorless solid; $[\alpha {]}_{D}^{25}$ −2.39 (c 0.67, MeOH); UV (MeOH) λ_max_ (log ε) 204 (3.4), 245 (1.6) nm; IR(film) ν_max_ 3391, 2931, 1753, 1681, 1452, 1380, 1189, 1095 cm^−1^; ^1^H and ^13^C data see Table 1; HR-ESIMS *m*/*z* 554.3107 [M + H]^+^, calcd. 554.3073 for C_32_H_44_NO_7_.
Aspochalasin A2 (**2**): colorless solid; $[\alpha {]}_{D}^{25}$ 8.08 (c 0.98, CHCl_3_); UV (MeOH) λ_max_ (log ε) 202 (3.6) nm; IR(KBr) ν_max_ 3454, 3273, 2924, 2852, 1709, 1435, 1384, 1324, 1263, 1142 cm^−1^; ^1^H and ^13^C data see Table 1; HR-ESIMS *m*/*z* 418.2589 [M + H]^+^, calcd. 418.2549 for C_24_H_36_NO_5_.
Aspochalasins D (**3**): colorless solid; UV (MeOH) λ_max_ (log ε) 204 (3.1) nm.
Aspergillulactone (**4**): colorless solid; UV (MeOH) λ_max_ (log ε) 204 (3.6), 245 (1.3) nm.
Aspochalasins T (**5**): colorless solid; UV (MeOH) λ_max_ (log ε) 205 (3.7) nm.

### 3.4. Preparation of MTPA Esters

Compound **1** was transferred into two clean NMR tubes (0.5 mg in each tube) and dried completely under vacuum. Deuterated pyridine (400 μL) was added to dissolve the sample and (R)-MTPA-Cl/(S)-MTPA-Cl (8μL) were quickly added into the tubes, respectively. All contents were mixed thoroughly by shaking the tubes carefully. The reaction was performed at room temperature for 4h to obtained the S-MTPA and the R-MTPA ester (**1a** and **1b**), respectively. The chemical shift differences (Δ*δ* = *δ_S_* − *δ_R_*) calculated from the ^1^H NMR spectra of the two diastereomeric esters **1a** and **1b**, obtained without purification, enabled us to determine the absolute configuration of C-17 as *S*.

Similarly, the S-MTPA and R-MTPA ester (**2a** and **2b**) of compound **2** was obtained. ^1^H NMR data of **2a** and **2b** were run without further purification and the chemical shift differences (Δ*δ* = *δ_S_* − *δ_R_*) enabled us to determine the absolute configuration of C-16 as *R*.

**1a:**^1^H NMR (500 MHz, pyridine-*d*_5_): *δ* 6.68 (1H, H-13), *δ* 1.37 (1H, H-16a), *δ* 7.86 (1H, H-17), *δ* 4.51 (1H, H-18), *δ* 2.77 (1H, H-19), *δ* 4.95 (1H, H-27a), *δ* 4.03 (1H, H-30), *δ* 4.32 (1H, H-32).
**1b:**^1^H NMR (500 MHz, pyridine-*d*_5_): *δ* 6.72 (1H, H-13), *δ* 1.42 (1H, H-16a), *δ* 7.92 (1H, H-17), *δ* 4.09 (1H, H-18), *δ* 2.71 (1H, H-19), *δ* 4.93 (1H, H-27a), *δ* 4.01 (1H, H-30), *δ* 4.25 (1H, H-32).
**2a:**^1^H NMR (500 MHz, pyridine-*d*_5_): *δ* 5.42 (1H, H-7), *δ* 3.26 (1H, H-8), *δ* 1.79 (3H, H-12), *δ* 6.61 (1H, H-13), *δ* 2.35 (1H, H-15a), *δ* 2.25 (1H, H-17a), *δ* 2.14 (1H, H-19a).
**2b:**^1^H NMR (500 MHz, pyridine-*d*_5_): *δ* 5.40 (1H, H-7), *δ* 3.24 (1H, H-8), *δ* 1.76 (3H, H-12), *δ* 6.55 (1H, H-13), *δ* 2.19 (1H, H-15a), *δ* 2.55 (1H, H-17a), *δ* 2.40 (1H, H-19a).

### 3.5. Cytotoxicity Bioassays

The cytotoxicity was measured by the MTT assay against the prostate cancer PC3 cell line. Tumor cell lines were seeded in 96-well plates (4 × 10^3^ per well in 100 μL). After 24 h of incubation, the cells were treated with gradient concentrations (100 μM, 50 μM, 25 μM, 12.5 μM, 6.25 μM, 3.125 μM) for another 72 h. Afterwards, MTT solution (5.0 mg/mL in RPMI-1640 media, Sigma, St. Louis, MO, USA) was added (20 μL/well) and the plates were incubated for another 4 h at 37 °C. The compounds were dissolved in DMSO and cell growth inhibition assay was performed as reported previously [26]. The growth inhibitory ability of the compounds were calculated and expressed using the IC_50_ value by dose-effect analysis software. Doxorubicin (ADR) was used as a positive control.

## 4. Conclusions

A chemical investigation was carried out on the marine fungus Z4, which resulted in the isolation of two novel aspochalasins (compounds **1** and **2**) along with three known aspochalasins (compounds **3**–**5**). The planar structures were determined and compound **1** contains a rare 5/6/6 tricyclic ring fused with the aspochalasin skeleton. Furthermore, the absolute configurations were established by modified Mosher’s method. Cytotoxicity against the prostate cancer PC3 cell line were assayed by MTT method. Compound **3** showed strong activity while others showed weak activity.

## Figures and Tables

**Table 1:** Nuclear magnetic resonance (NMR) spectroscopic data of compounds **1** and **2** in CDCl_3_.

Pos.	1		2	
	*δ*_C_^a^, Type	*δ*_H_ (*J* in Hz) ^b^	*δ*_C_^a^, Type	*δ*_H_ (*J* in Hz) ^b^
1	176.4, C		172.4, C	
2		6.10, brs		5.96, brs
3	52.9, CH	2.97, m	52.4, CH	2.99, m
4	53.9, CH	2.81, t (4.4)	52.3, CH	2.56, dd (8.5,7.1)
5	35.9, CH	2.67, brs	34.8, CH	2.80, brs
6	140.6, C		140.9, C	
7	125.6, CH	5.45, brs	123.5, CH	5.29, brs
8	42.9, CH	3.61, d (12.1)	41.1, CH	3.36, d (10.3)
9	68.9, C		86.5, C	
10	47.2, CH_2_	1.49, m; 1.25, m	46.8, CH_2_	1.83, m; 1.23, m
11	14.4, CH_3_	1.18, d (7.1)	14.3, CH_3_	1.19, d (7.3)
12	20.5, CH_3_	1.77, s	20.1, CH_3_	1.77, s
13	125.1, CH	5.70, d (11.4)	123.8, CH	5.94, d (10.3)
14	135.9, C		138.2, C	
15	36.9, CH_2_	2.07, m	33.1, CH_2_	2.27, m; 2.14, m
16	29.3, CH	1.50, m; 1.30, m	75.1, CH	4.42, brs
17	68.6, CH	4.35, d (6.3)	32.3, CH_2_	2.04, m; 2.28, m
18	88.8, CH	3.86, d (11.3)	208.9, C	
19	44.0, CH	2.25, m	29.0, CH_2_	2.32, m; 3.02, m
20	47.7, CH	3.51, dd (4.9, 11.0)	34.1, CH_2_	2.62, m; 2.44, m
21	214.2, C		171.9, C	
22	26.0, CH	1.53, m	25.6, CH	1.59, m
23	21.2, CH_3_	0.93, d (6.4)	21.1, CH_3_	0.92, d (6.5)
24	24.2, CH_3_	0.96, d (6.4)	23.8, CH_3_	0.94, d (6.5)
25	16.1, CH_3_	1.57, s	17.7, CH_3_	1.56, s
26	109.4, CH	5.91, d (3.9)		
27	73.1, CH_2_	4.79, m		
28	158.1, C			
29	127.6, C			
30	51.4, CH	3.59, m		
31	197.0, C			
32	77.3, CH	4.42, dd (3.6, 11.1)		
33	14.0, CH_3_	1.85, s		

^a^ Recorded at 500 MHz in CDCl_3_. ^b^ Recorded at 125 MHz in CDCl_3_.

**Table 2:** Growth inhibition of **1**–**5** against prostate cancer PC3 cell line.

Compounds	PC3 Cell Line (IC_50_ in μM)
1	>36
2	>40
3	11.14
4	>40
5	>40
ADR	5.09

## Supplementary Materials

The following are available online at http://www.mdpi.com/1660-3397/16/10/343/s1, Figure S1: IR spectrum of **1**; Figures S2 and S3: HR-ESI-MS spectrum of **1**; Figures S4–S10: ^1^H, ^13^C, DEPT, HSQC, COSY, HMBC, NOESY NMR data of **1** in CDCl_3_; Figures S11 and S12: ^1^H NMR data of **1a** and **1b** in pyridine-*d*_5_; Figure S13: IR spectrum of **2**; Figures S14 and S15: HR-ESI-MS spectrum of **2**; Figures S16–S22: ^1^H, ^13^C, DEPT, HSQC, COSY, HMBC, NOESY NMR data of **2** in CDCl_3_; Figures S23 and S24: ^1^H NMR data of **2a** and **2b** in pyridine-*d*_5_; Figures S25 and S26: ^1^H and ^13^C NMR data of **3** in CDCl_3_; Figures S27 and S28: ^1^H and ^13^C NMR data of **4** in CDCl_3_; Figures S29 and S30: ^1^H and ^13^C NMR data of **5** in CDCl_3_.

## References

[1] Scherlach K., Boettger D., Remme N., Hertweck C. (2010). The chemistry and biology of cytochalasans. Nat. Prod. Rep..

[2] Zhang D., Ge H., Xie D., Chen R., Zou J.H., Tao X., Dai J. (2013). Periconiasins A–C, new cytotoxic cytochalasans with an unprecedented 9/6/5 tricyclic ring system from endophytic fungus *periconia* sp.. Org. Lett..

[3] Knudsen P.B., Hanna B., Ohl S., Sellner L., Zenz T., Dohner H., Stilgenbauer S., Larsen T.O., Lichter P., Seiffert M. (2014). Chaetoglobosin A preferentially induces apoptosis in chronic lymphocytic leukemia cells by targeting the cytoskeleton. Leukemia.

[4] Zhou G.X., Wijeratne E.M.K., Bigelow D., Pierson L.S., Vanetten H.D., Guantilaka A.A.L. (2004). Aspochalasins I, J, and K: Three new cytotoxic cytochalasans of *Aspergillus flavipes* from the rhizosphere of *Ericameria laricifolia* of the sonoran desert. J. Nat. Prod..

[5] Rochfort S., Ford J., Ovenden S., Wan S.S., George S., Wildman H., Tait R.M., Meurer-Grimes B., Cox S., Coates J. (2005). A novel aspochalasin with HIV-1 integrase inhibitory activity from *Aspergillus flavipes*. J. Antibiot..

[6] Hua C.Y., Yang Y.H., Sun L., Dou H., Tan R.X., Hou Y.Y. (2013). Chaetoglobosin F, a small molecule compound, possesses immunomodulatory properties on bone marrow-derived dendritic cells via TLR9 signaling pathway. Immunobiology.

[7] Hu Y., Zhang W.P., Zhang P., Ruan W.B., Zhu X.D. (2013). Nematicidal Activity of Chaetoglobosin A Poduced by *Chaetomium globosum* NK102 against Meloidogyne incognita. J. Agric. Food Chem..

[8] Qiao K.J., Chooi Y.H., Tang Y. (2011). Identification and engineering of the cytochalasin gene cluster from Aspergillus clavatus NRRL 1. Metab. Eng..

[9] Schumann J., Hertweck C. (2007). Molecular basis of cytochalasan biosynthesis in fungi: Gene cluster analysis and evidence for the involvement of a PKS-NRPS hybrid synthase by RNA silencing. J. Am. Chem. Soc..

[10] Oh H., Swenson D.C., Gloer J.B., Wicklow D.T., Dowd P.F. (1998). Chaetochalasin A: A new bioactive metabolite from *Chaetomium brasiliense*. Tetrahedron. Lett..

[11] Zhang Y., Wang T., Pei Y.H., Hua H.M., Feng B.M. (2002). Aspergillin PZ, a novel isoindole-alkaloid from *Aspergillus awamori*. J. Antibiot..

[12] Lin Z.J., Zhu T.J., Wei H.J., Zhang G.J., Wang H., Gu Q.Q. (2009). Spicochalasin A and New Aspochalasins from the Marine-Derived Fungus *Spicaria elegans*. Eur. J. Org. Chem..

[13] Zhu H.C., Chen C.M., Tong Q.Y., Li X.N., Yang J., Xue Y.B., Luo Z.W., Wang J.P., Yao G.M., Zhang Y.H. (2016). Epicochalasines A and B: Two bioactive merocytochalasans bearing caged epicoccine dimer units from *Aspergillus flavipes*. Angew. Chem. Int. Ed..

[14] Li X., Zhao Z., Ding W., Ye B., Wang P., Xu J. (2017). Aspochalazine A, a novel polycyclic aspochalasin from the fungus *Aspergillus* sp. Z4. Tetrahedron Lett..

[15] Liu Y., Zhao S., Ding W., Wang P., Yang X., Xu J. (2014). Methylthio-aspochalasins from a marine-derived fungus *Aspergillus* sp.. Mar. Drugs.

[16] Xu J.Z., Zhao S.Z., Yang X.W. (2014). A new cyclopeptide metabolite of marine gut fungus from *Ligia oceanica*. Nat. Prod. Res..

[17] Wang P.M., Zhao S.Z., Liu Y., Ding W.J., Qiu F., Xu J.Z. (2015). Asperginine, an unprecedented alkaloid from the marine-derived fungus *Aspergillus* sp.. Nat. Prod. Commun..

[18] Wang T., Zhang Y., Pei Y.h. (2007). Two novel trichothecenes from *Myrothecium roridum*. Med. Chem. Res..

[19] Ding G., Wang H., Li L., Chen A.J., Chen L., Chen H., Zhang H., Liu X., Zou Z. (2012). Trichoderones A and B: Two pentacyclic cytochalasans from the plant endophytic fungus *Trichoderma gamsii*. Eur. J. Org. Chem..

[20] Liu R., Gu Q., Zhu W., Cui C., Fan G., Fang Y., Zhu T., Liu H. (2006). 10-Phenyl-[12]-cytochalasins Z7, Z8, and Z9 from the marine-derived fungus *Spicaria elegans*. J. Nat. Prod..

[21] Chen C., Tong Q., Zhu H., Tan D., Zhang J., Xue Y., Yao G., Luo Z., Wang J., Wang Y. (2016). Nine new cytochalasan alkaloids from Chaetomium globosum TW1-1 (Ascomycota, Sordariales). Sci. Rep..

[22] Zheng C.J., Shao C.L., Wu L.Y., Chen M., Wang K.L., Zhao D.L., Sun X.P., Chen G.Y., Wang C.Y. (2013). Bioactive phenylalanine derivatives and cytochalasins from the soft coral-derived fungus, *Aspergillus elegans*. Mar. Drugs.

[23] Tomikawa T., Shin-ya K., Kinoshita T., Miyajima A., Seto H., Hayakawa Y. (2001). Selective cytotoxicity and stereochemistry of aspochalasin D. J. Antibiot..

[24] Rukachaisirikul V., Rungsaiwattana N., Klaiklay S., Phongpaichit S., Borwornwiriyapan K., Sakayaroj J. (2014). γ-Butyrolactone, cytochalasin, cyclic carbonate, eutypinic acid, and phenalenone derivatives from the soil fungus *Aspergillus* sp. PSU-RSPG185. J. Nat. Prod..

[25] Lin Z.J., Zhu T.J., Chen L., Gu Q.Q. (2010). Three new aspochalasin derivatives from the marine-derived fungus *Spicaria elegans*. Chin. Chem. Lett..

[26] Wang F., Hua H.M., Pei Y.H., Chen D., Jing Y.K. (2006). Triterpenoids from the resin of *Styrax tonkinensis* and their antiproliferative and differentiation effects in human leukemia HL-60 cells. J. Nat. Prod..

