# Supplementary material for: Two Novel Aspochalasins from the Gut Fungus Aspergillus sp. Z4
Source: Mar Drugs. 2018 Sep 20;16(10):343. doi: 10.3390/md16100343 (PMC6213381; doi:10.3390/md16100343)
Supplement: Supplementary file 1 [file marinedrugs-16-00343-s001.pdf]

marine drugs

Two Novel Aspochalasins From the Gut Fungus *Aspergillus* sp. Z4

Xinyang Li [1], Wanjing Ding [1], Pinmei Wang [1] and Jinzhong Xu [1,*]

1 Institute of Marine Biology, Ocean College, Zhejiang University, Zhoushan 316021, China
* Correspondence: xujinzhong@zju.edu.cn; Tel.: +86 15858168018

Contents

Figure S1. The IR spectrum of compound **1**......3
Figure S2. The HR-ESI-MS spectrum of compound **1** 3
Figure S3. The HR-ESI-MS spectrum of compound **1** (expansion)......4
Figure S4. The ^{1}H-NMR spectrum of compound **1** in $CDCl_3$ 4
Figure S5. The ^{13}C-NMR spectrum of compound **1** in $CDCl_3$......5
Figure S6. The DEPT spectrum of compound **1** in $CDCl_3$......5
Figure S7. The HSQC spectrum of compound **1** in $CDCl_3$......6
Figure S8. The ^{1}H-^{1}H COSY spectrum of compound **1** in $CDCl_3$......6
Figure S9. The HMBC spectrum of compound **1** in $CDCl_3$......7
Figure S10. The NOESY spectrum of compound **1** in $CDCl_3$ 7
Figure S11. The ^{1}H spectrum of compound **1a** in pyridine-d_5......8
Figure S12. The ^{1}H spectrum of compound **1b** in pyridine-d_5......8
Figure S13. The IR spectrum of compound **2**......9
Figure S14. The HR-ESI-MS spectrum of compound **2** 9
Figure S15. The HR-ESI-MS spectrum of compound **2** (expansion)......10
Figure S16. The ^{1}H-NMR spectrum of compound **2** in $CDCl_3$ 10
Figure S17. The ^{13}C-NMR spectrum of compound **2** in $CDCl_3$......11
Figure S18. The DEPT spectrum of compound **2** in $CDCl_3$......11
Figure S19. The HSQC spectrum of compound **2** in $CDCl_3$......12
Figure S20. The ^{1}H-^{1}H COSY spectrum of compound **2** in $CDCl_3$......12
Figure S21. The HMBC spectrum of compound **2** in $CDCl_3$......13
Figure S22. The NOESY spectrum of compound **2** in $CDCl_3$ 13
Figure S23. The ^{1}H spectrum of compound **2a** in pyridine-d_5......14
Figure S24. The ^{1}H spectrum of compound **2b** in pyridine-d_5......14
Figure S25. The ^{1}H-NMR spectrum of compound **3** in $CDCl_3$ 15
Figure S26. The ^{13}C-NMR spectrum of compound **3** in $CDCl_3$......15
Figure S27. The ^{1}H-NMR spectrum of compound **4** in $CDCl_3$ 16
Figure S28. The ^{13}C-NMR spectrum of compound **4** in $CDCl_3$......16
Figure S29. The ^{1}H-NMR spectrum of compound **5** in $CDCl_3$ 17
Figure S30. The ^{13}C-NMR spectrum of compound **5** in $CDCl_3$......17

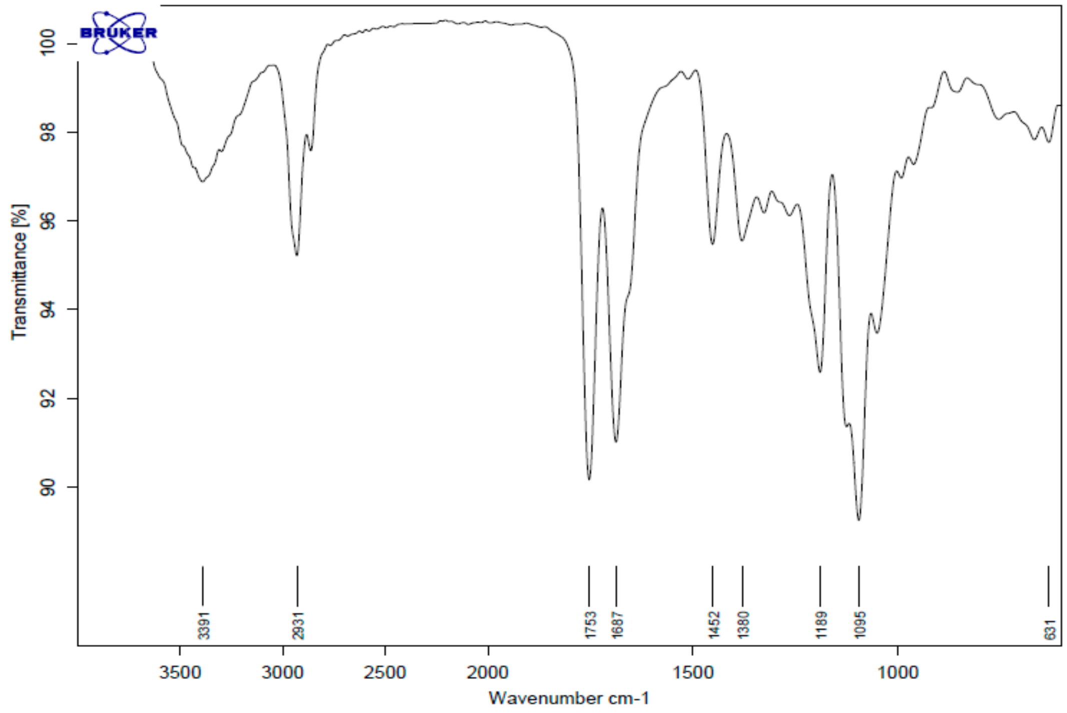

Figure S1. The IR spectrum of compound **1**

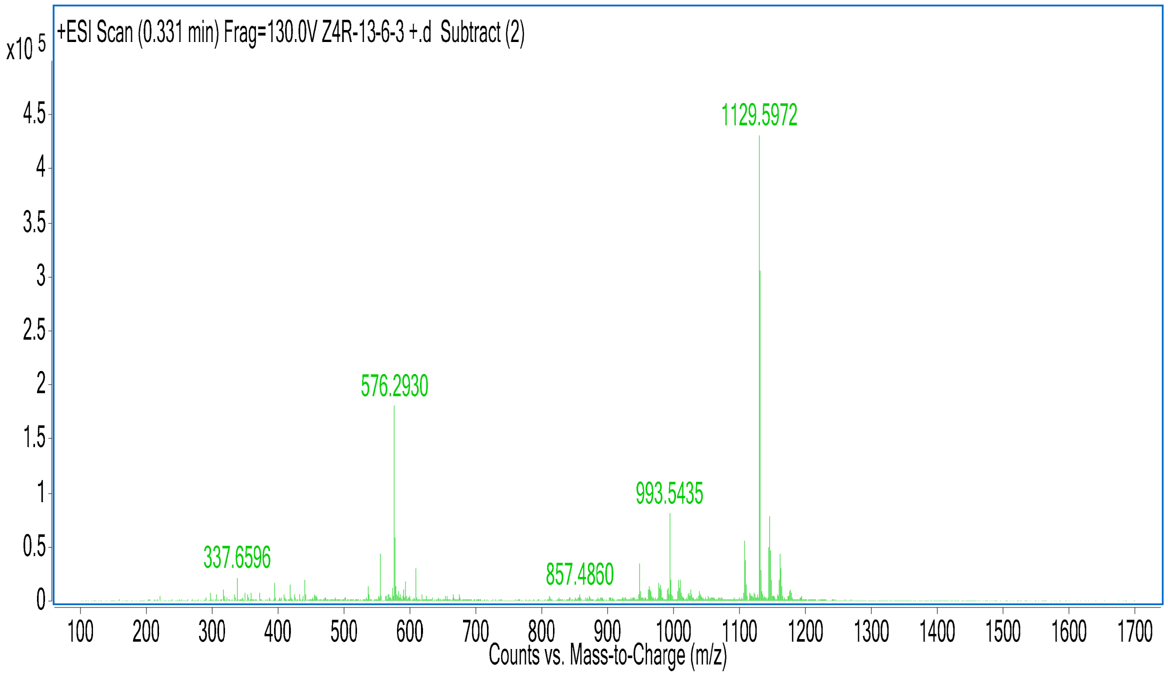

Figure S2. The HR-ESI-MS spectrum of compound **1**

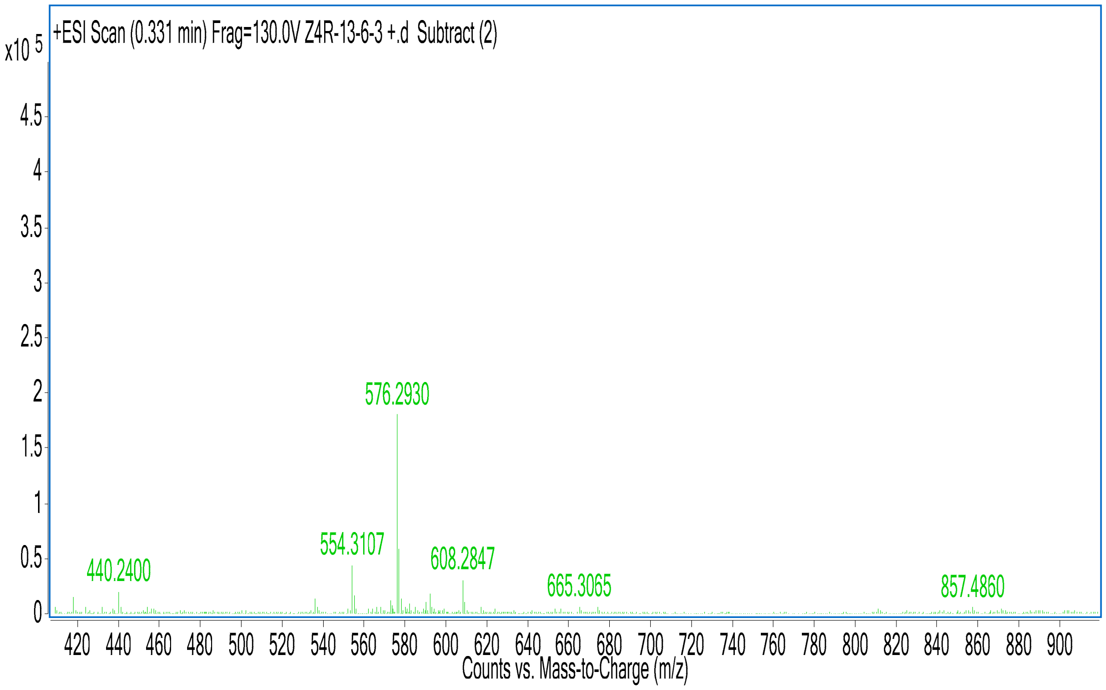

Figure S3. The HR-ESI-MS spectrum of compound **1** (expansion)

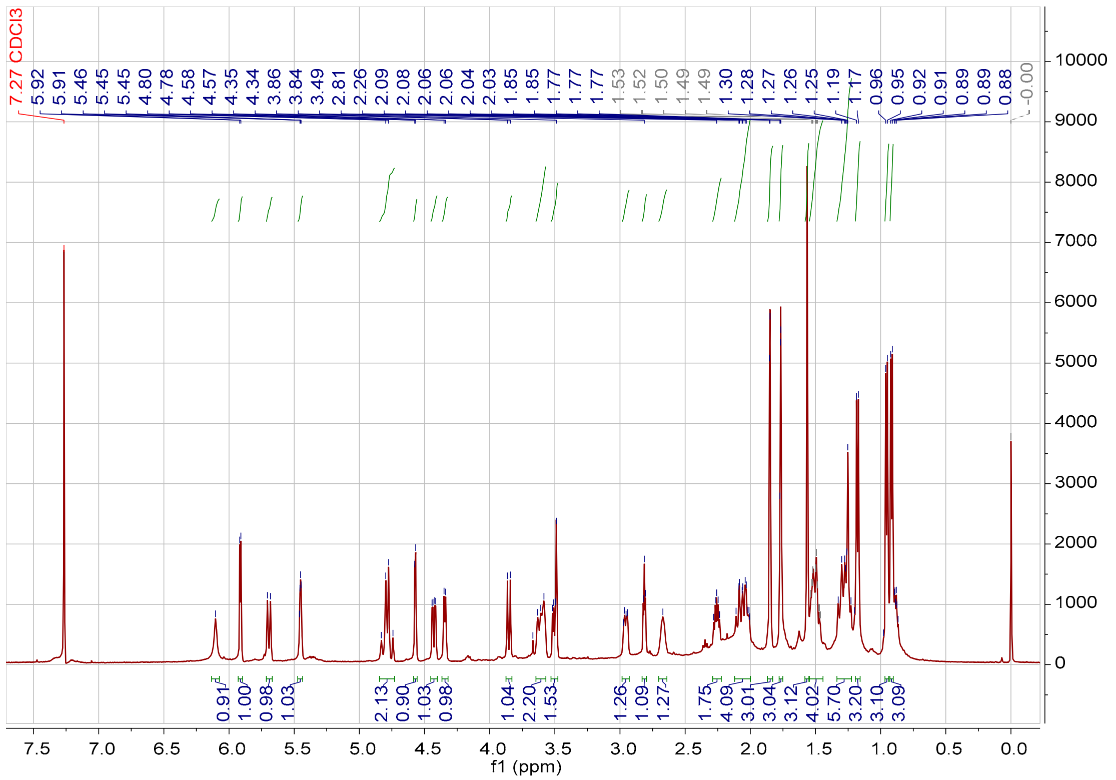

Figure S4. The ^{1}H-NMR spectrum of compound **1** in $CDCl_3$

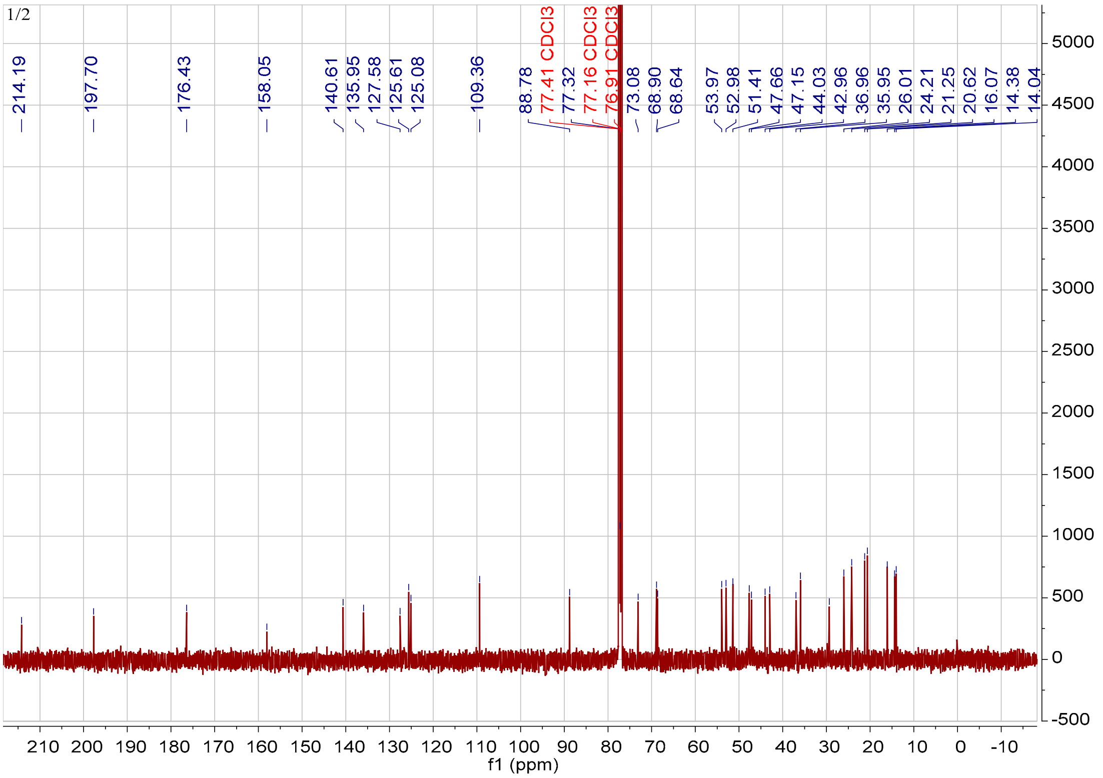

Figure S5. The ^{13}C-NMR spectrum of compound **1** in $CDCl_3$

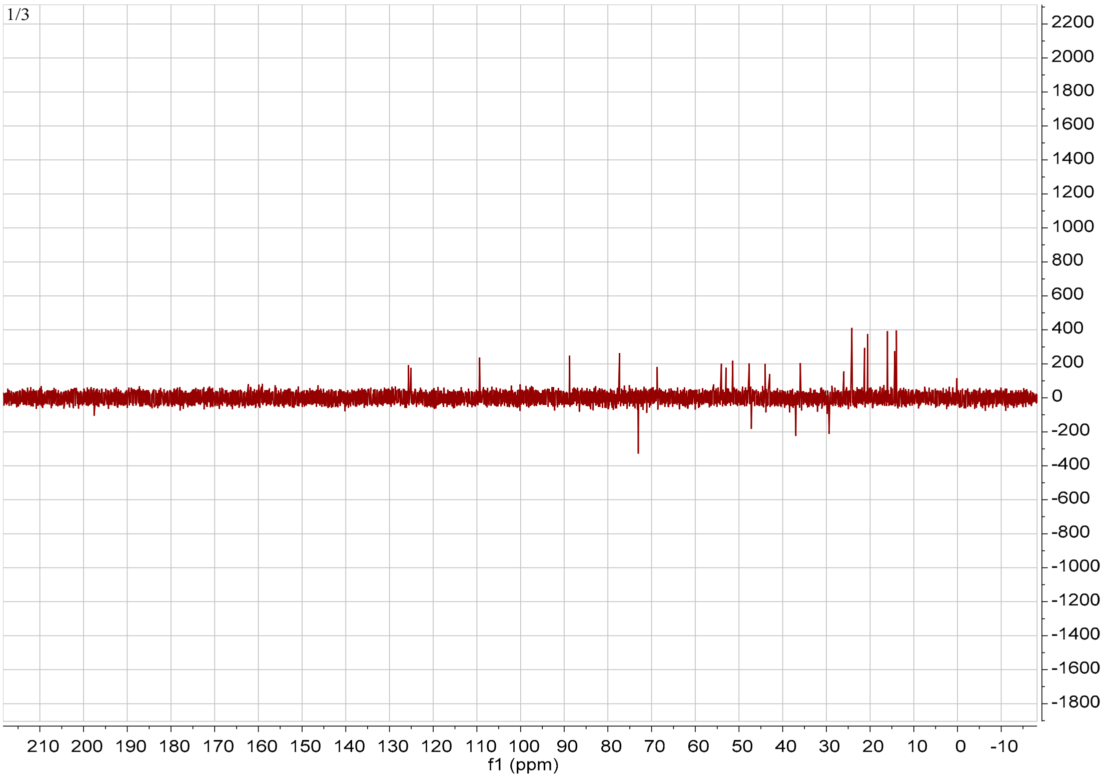

Figure S6. The DEPT spectrum of compound **1** in $CDCl_3$

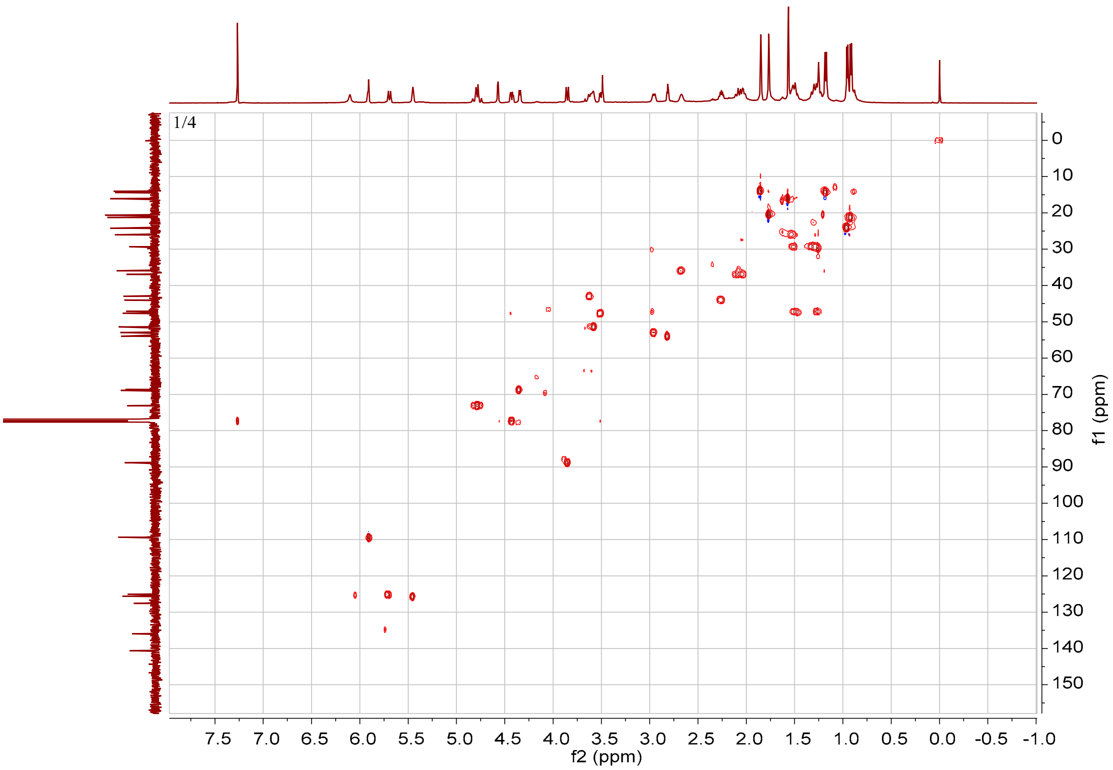

Figure S7. The HSQC spectrum of compound **1** in $CDCl_3$

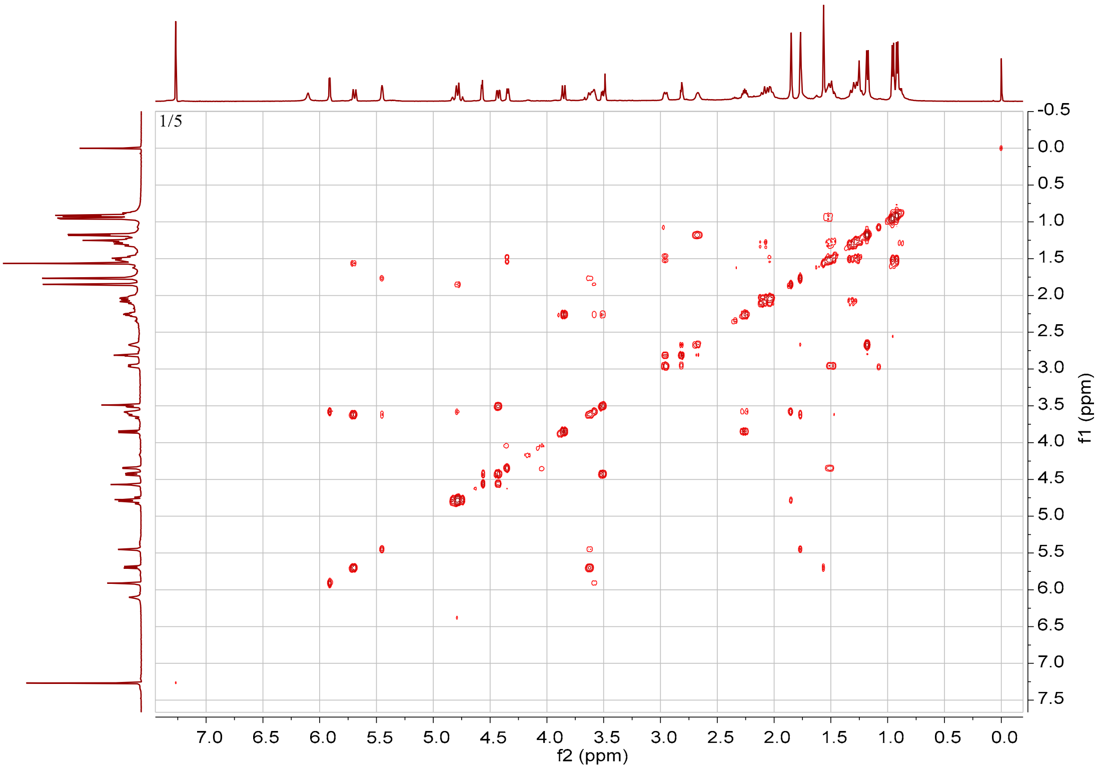

Figure S8. The ^{1}H-^{1}H COSY spectrum of compound **1** in $CDCl_3$

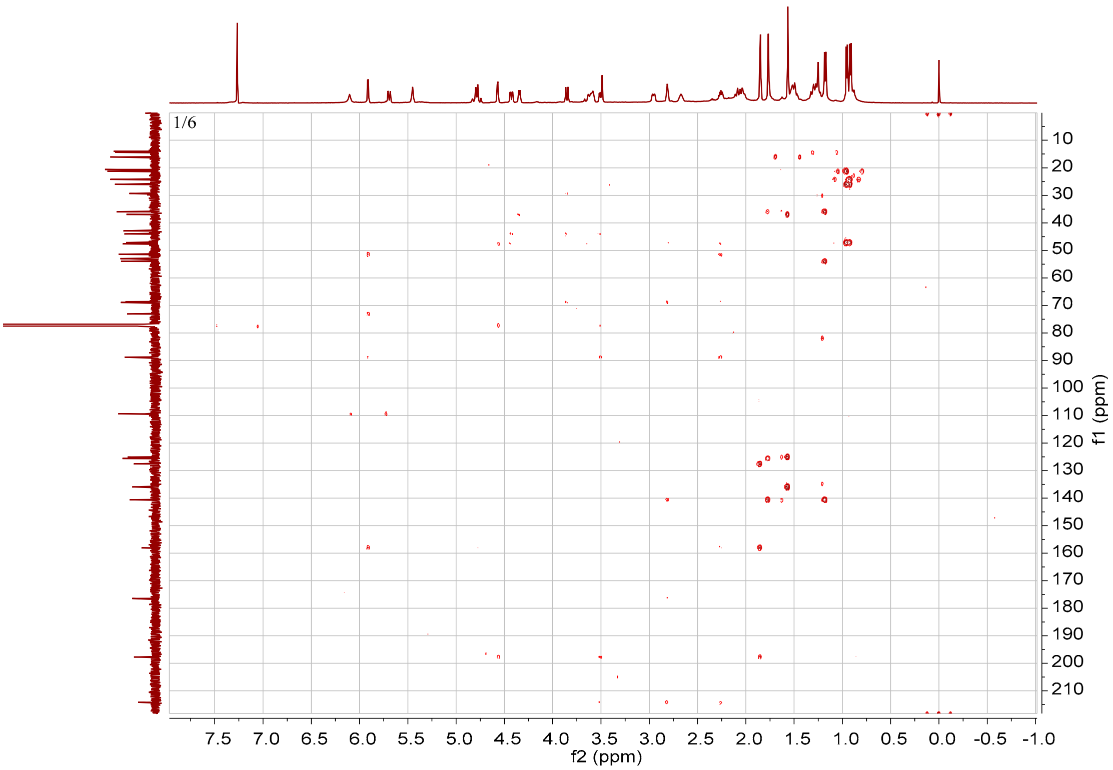

Figure S9. The HMBC spectrum of compound **1** in $CDCl_3$

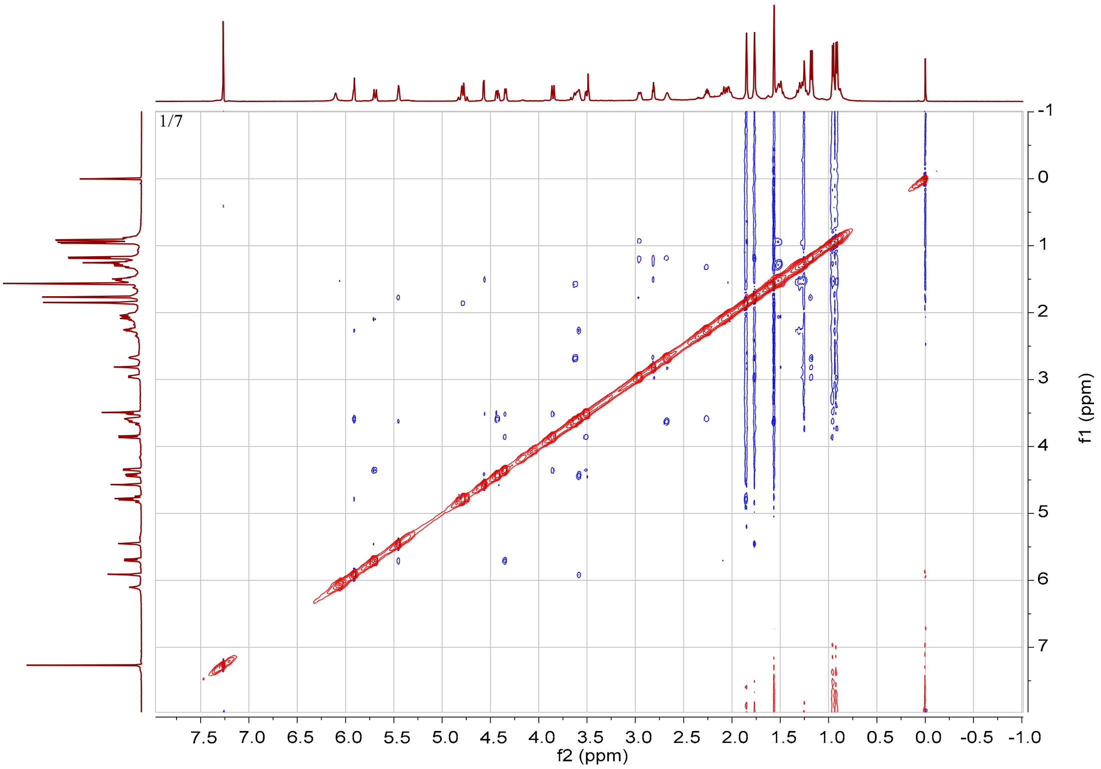

Figure S10. The NOESY spectrum of compound **1** in $CDCl_3$

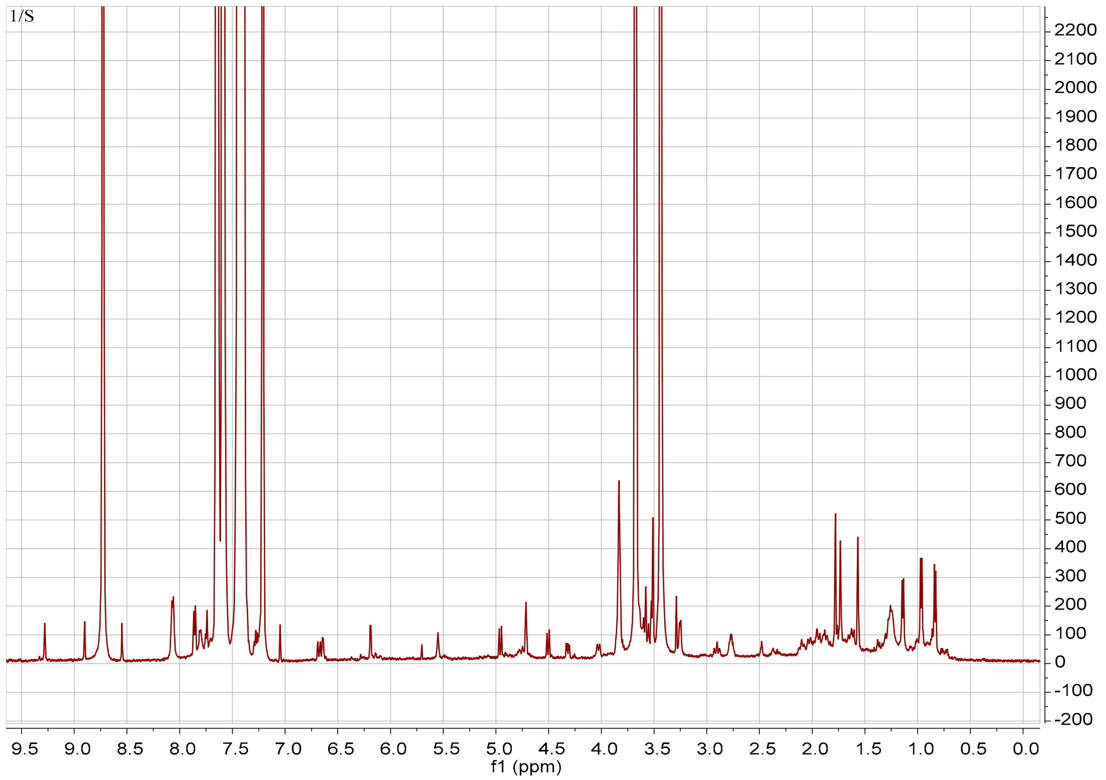

Figure S11. The ^{1}H spectrum of compound **1a** in pyridine-d_5

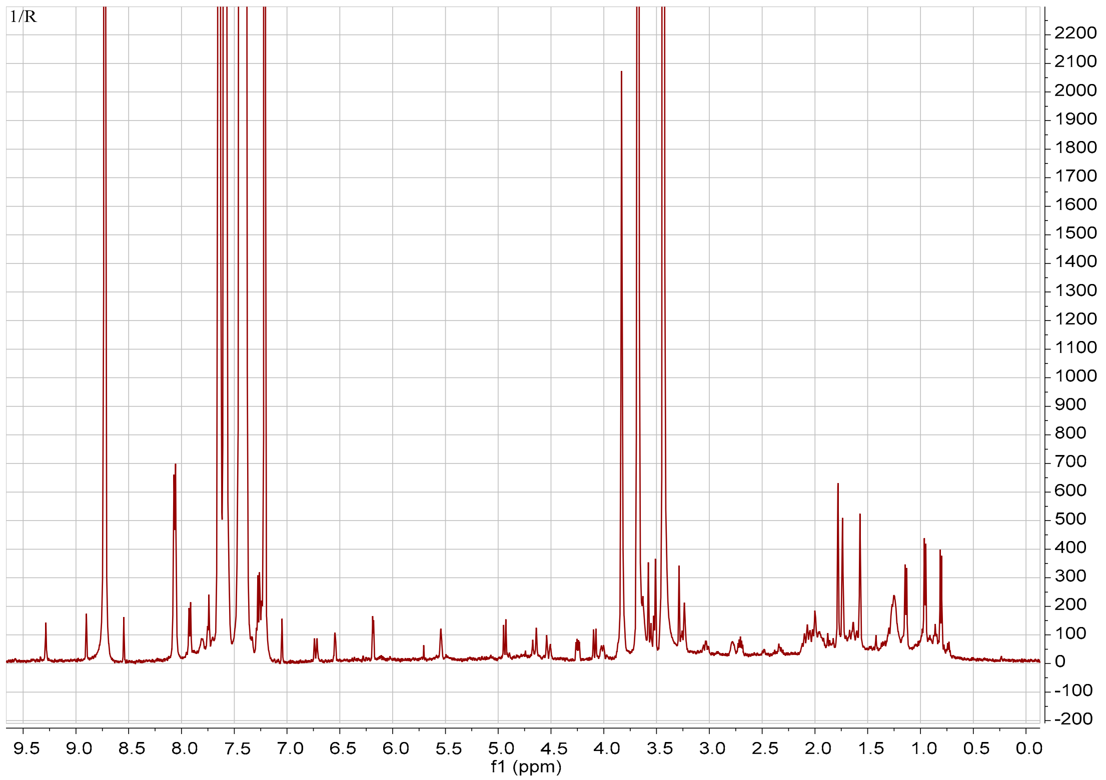

Figure S12. The ^{1}H spectrum of compound **1b** in pyridine-d_5

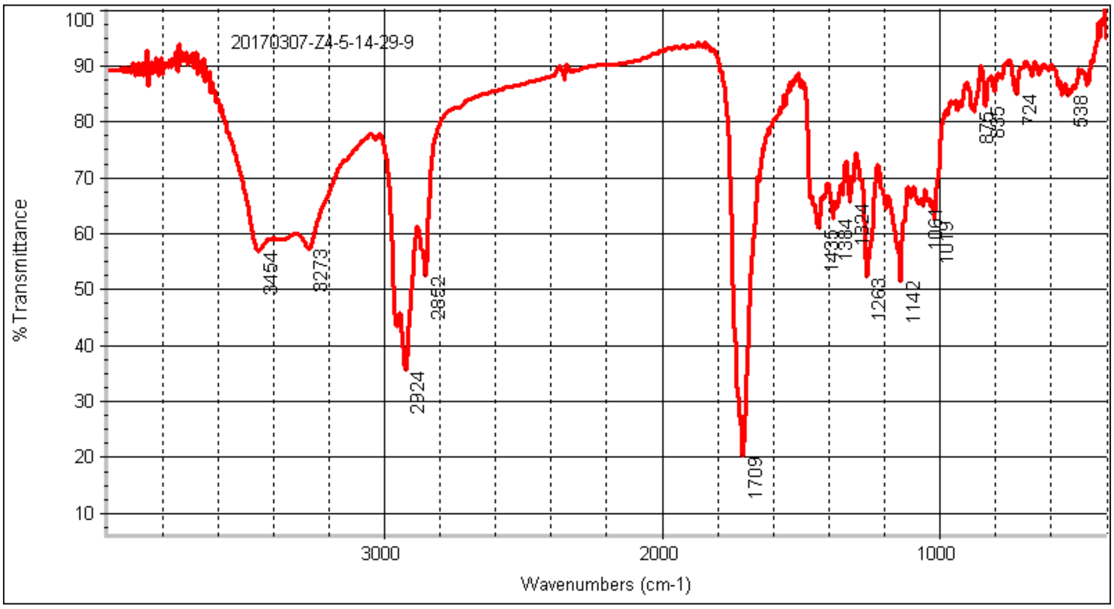

Figure S13. The IR spectrum of compound **2**

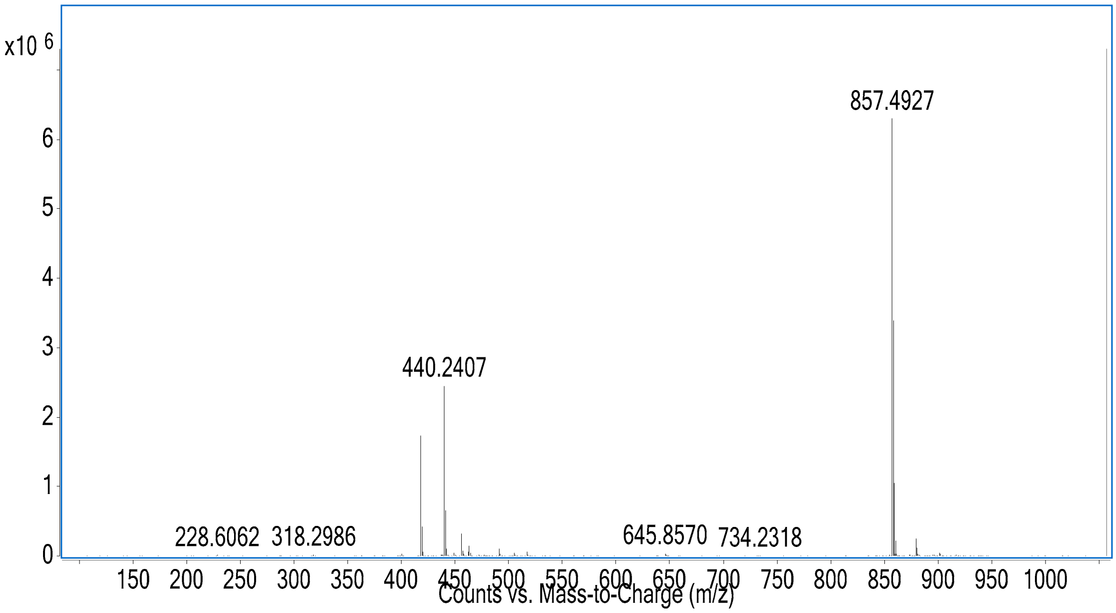

Figure S14. The HR-ESI-MS spectrum of compound **2**

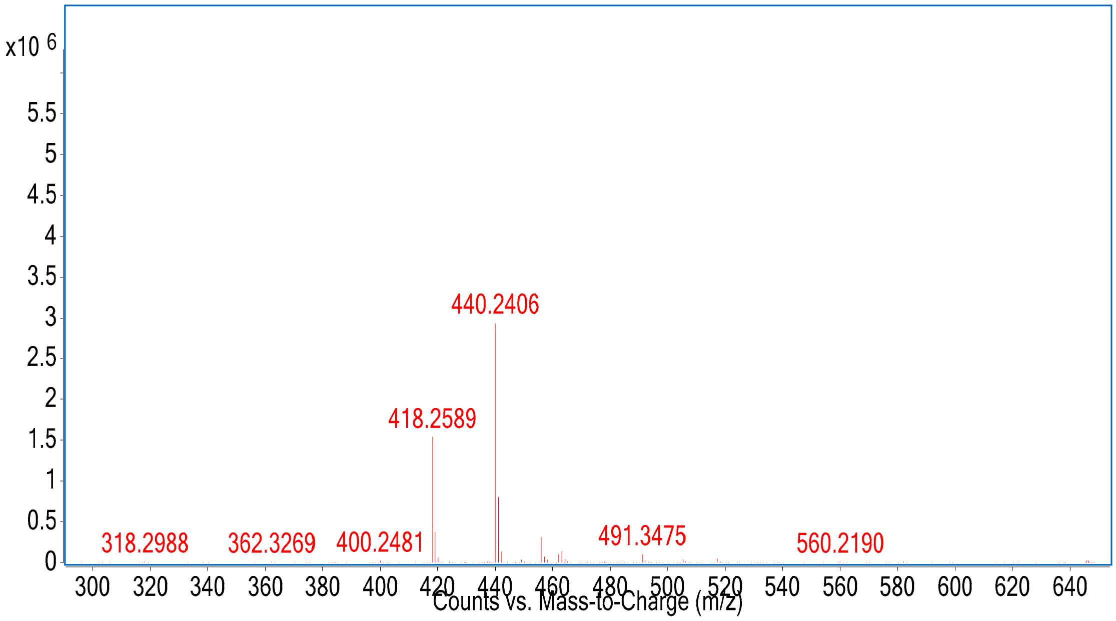

Figure S15. The HR-ESI-MS spectrum of compound **2** (expansion)

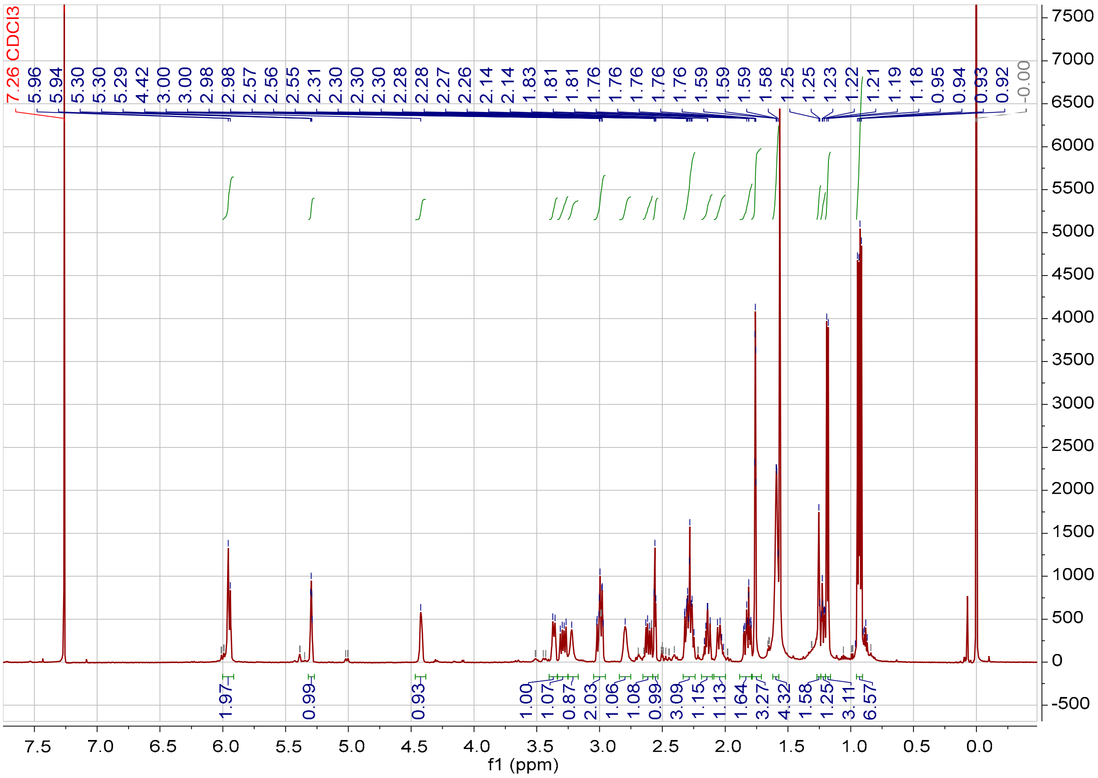

Figure S16. The ^{1}H-NMR spectrum of compound **2** in $CDCl_3$

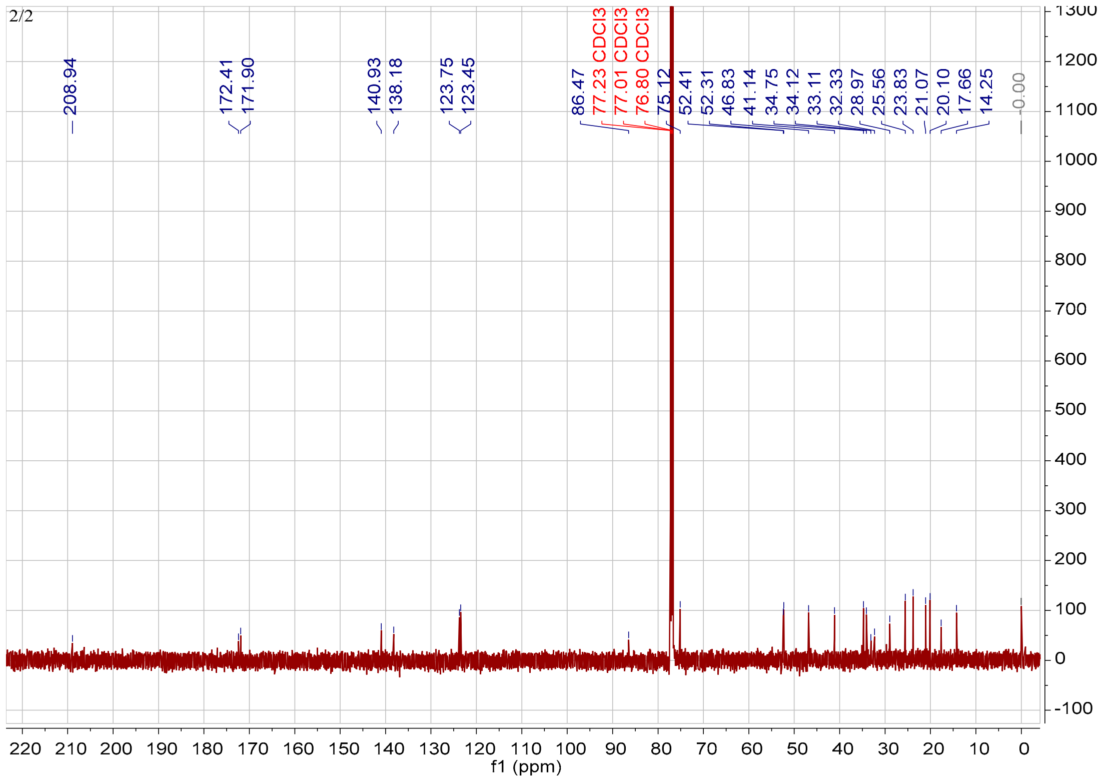

Figure S17. The ^{13}C-NMR spectrum of compound **2** in $CDCl_3$

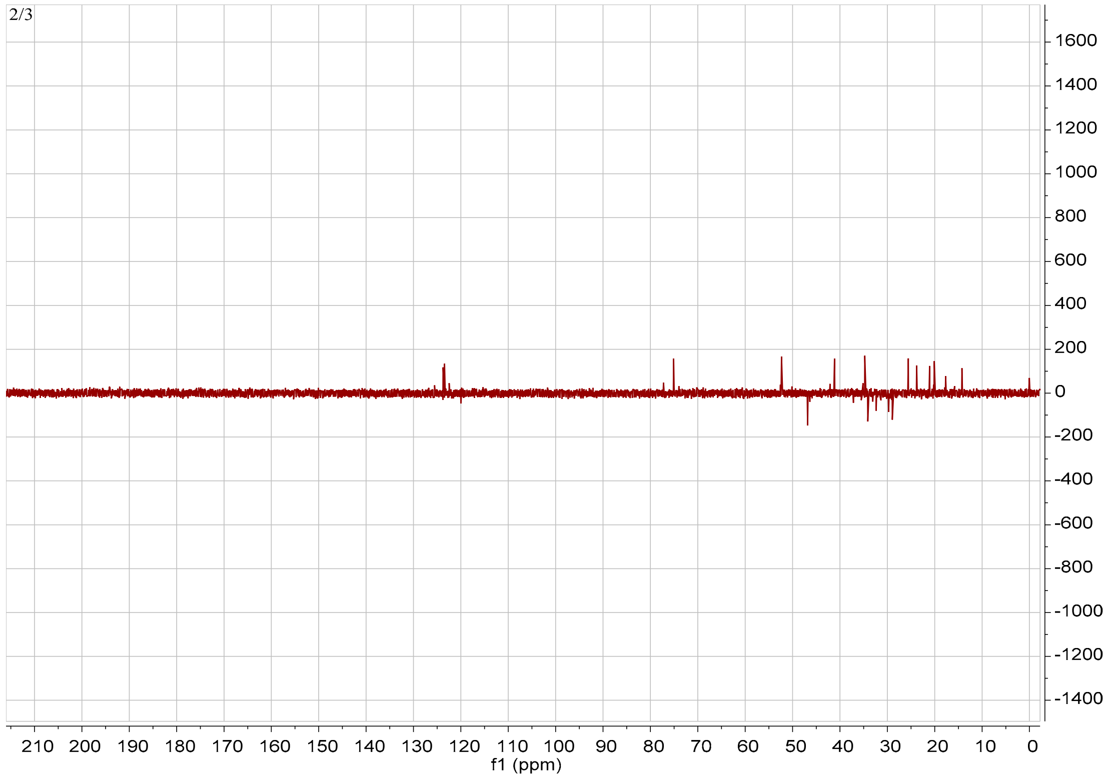

Figure S18. The DEPT spectrum of compound **2** in $CDCl_3$

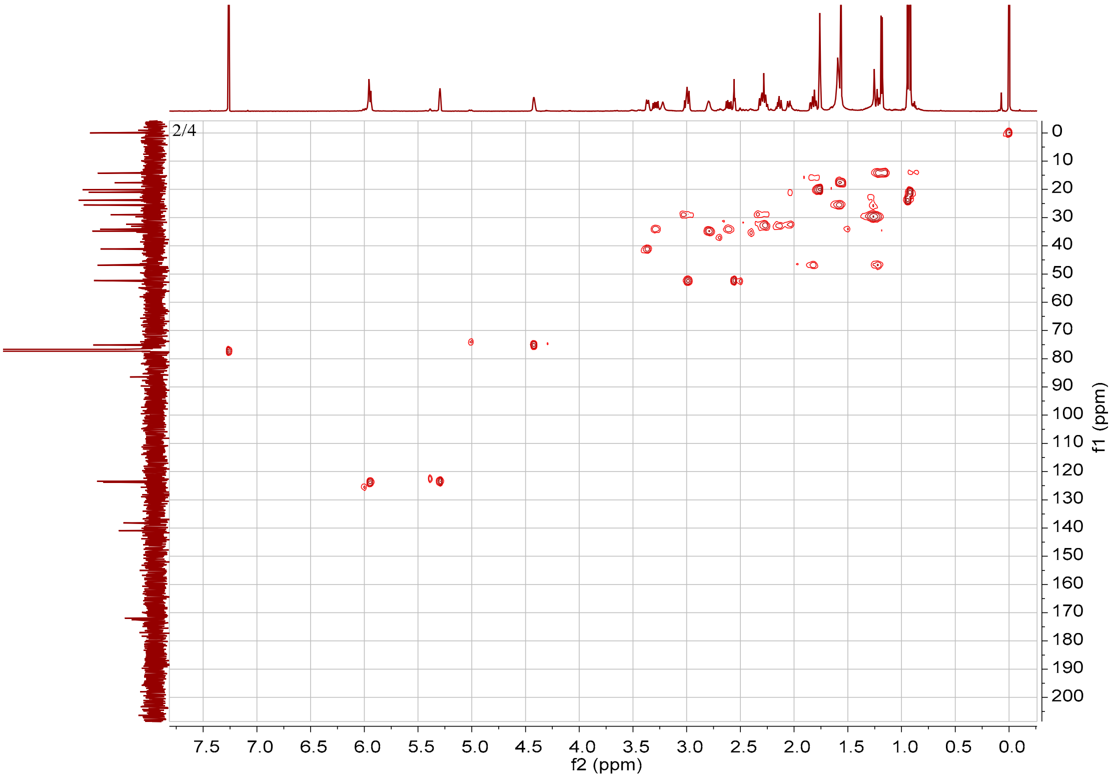

Figure S19. The HSQC spectrum of compound **2** in $CDCl_3$

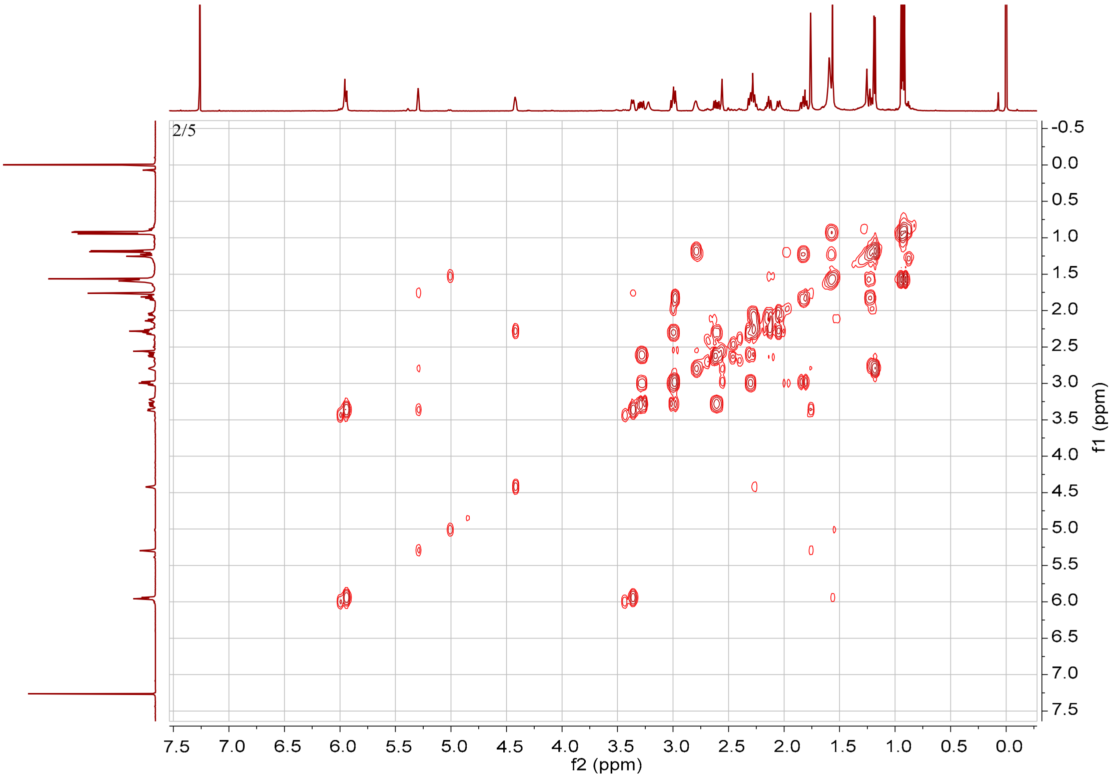

Figure S20. The ^1H-^1H COSY spectrum of compound **2** in $CDCl_3$

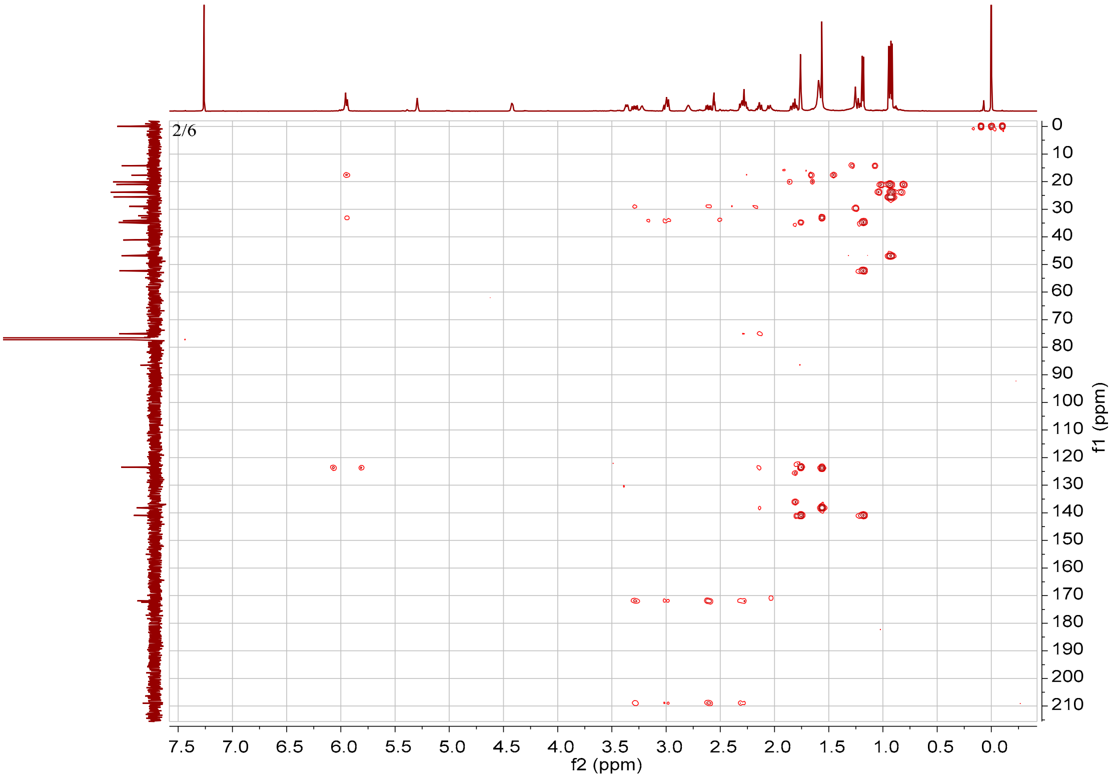

Figure S21. The HMBC spectrum of compound **2** in $CDCl_3$

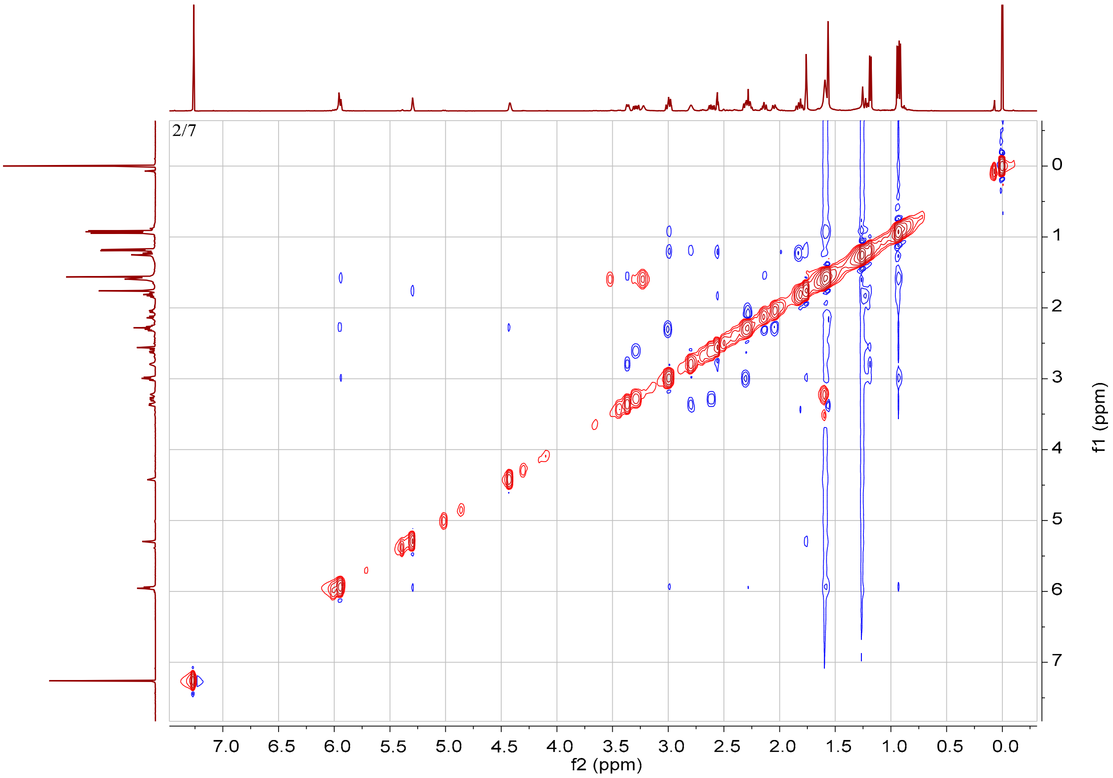

Figure S22. The NOESY spectrum of compound **2** in $CDCl_3$

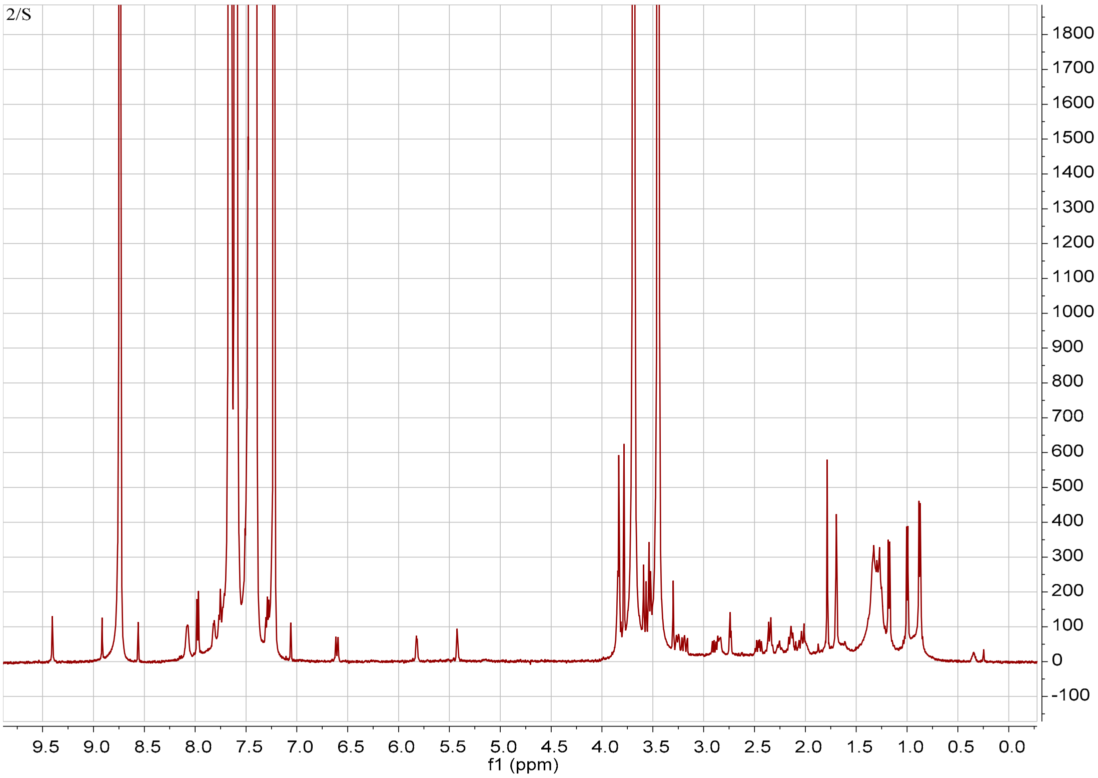

Figure S23. The ^{1}H spectrum of compound **2a** in pyridine-*d*5

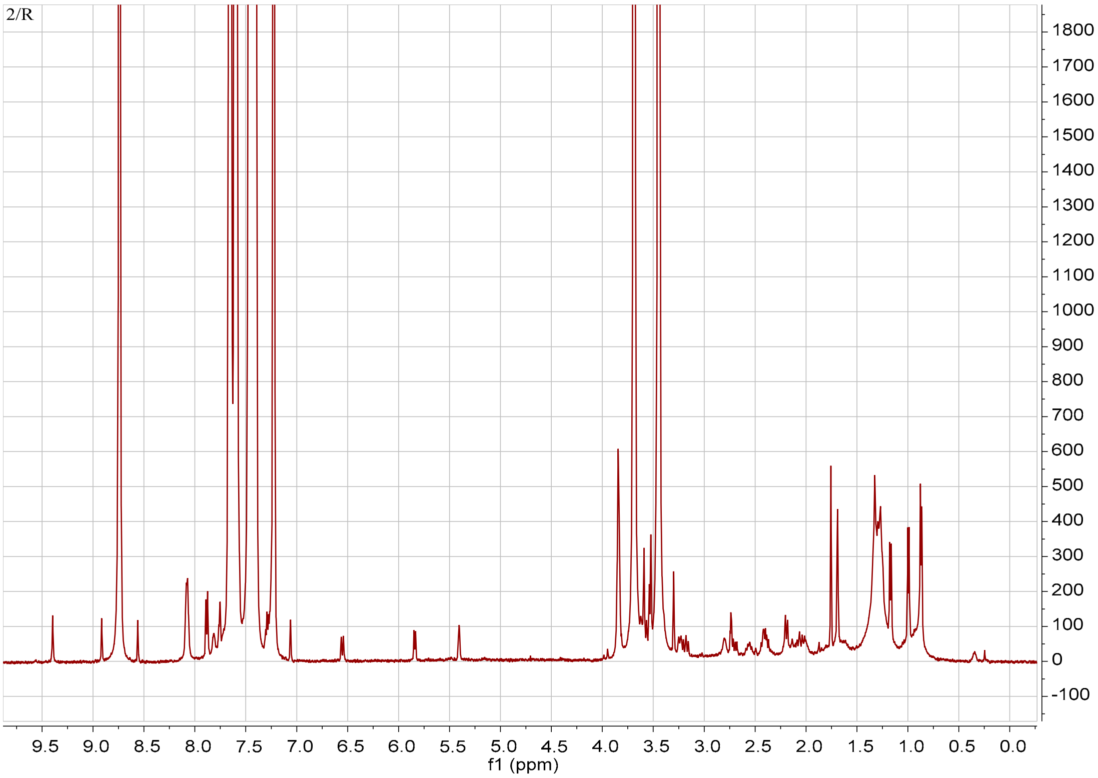

Figure S24. The ^{1}H spectrum of compound **2b** in pyridine-*d*5

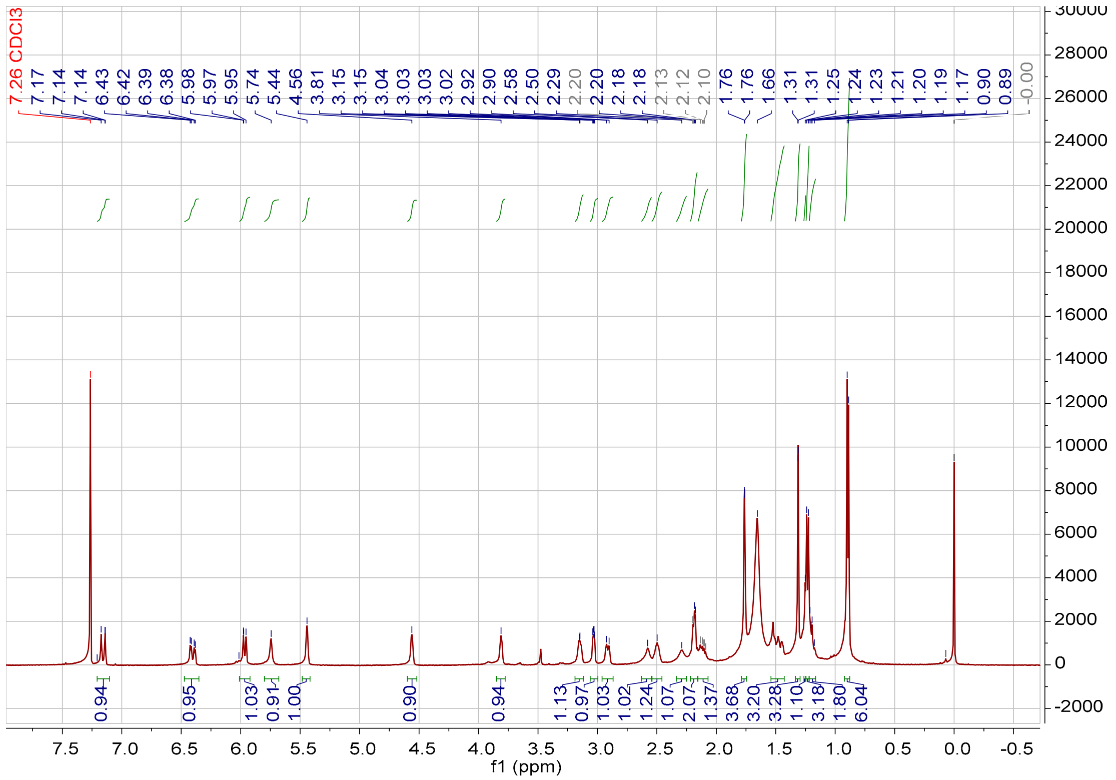

Figure S25. The ^{1}H-NMR spectrum of compound **3** in $CDCl_3$

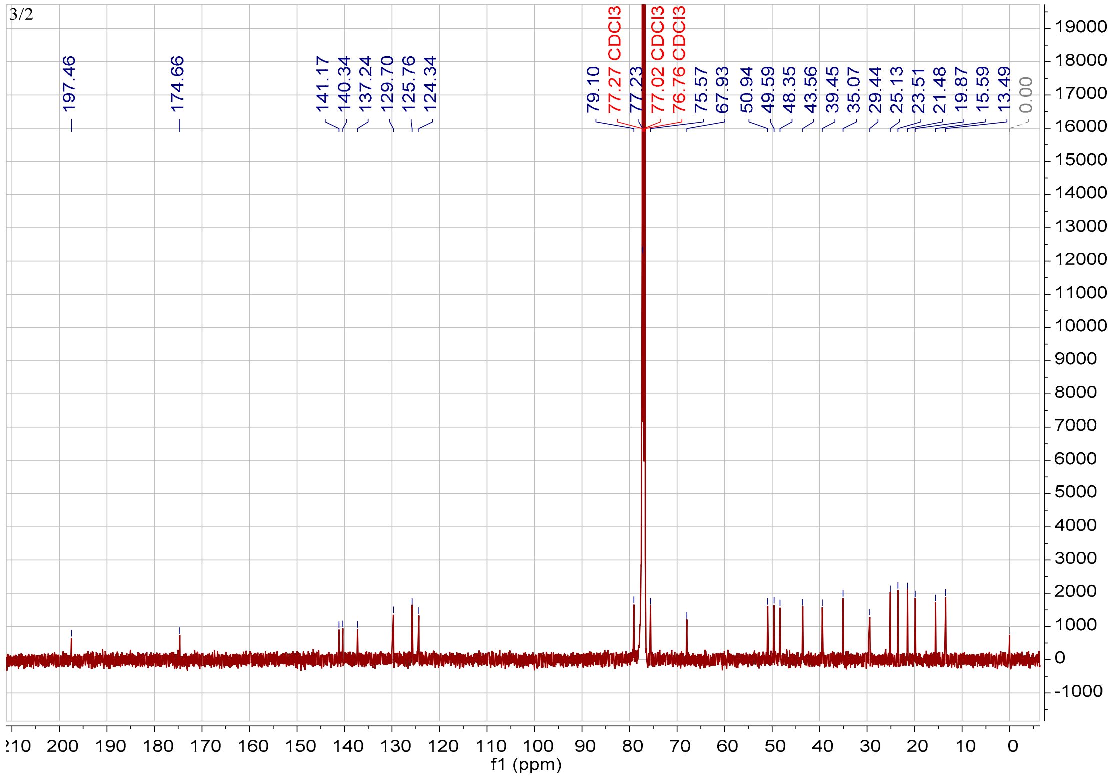

Figure S26. The ^{13}C-NMR spectrum of compound **3** in $CDCl_3$

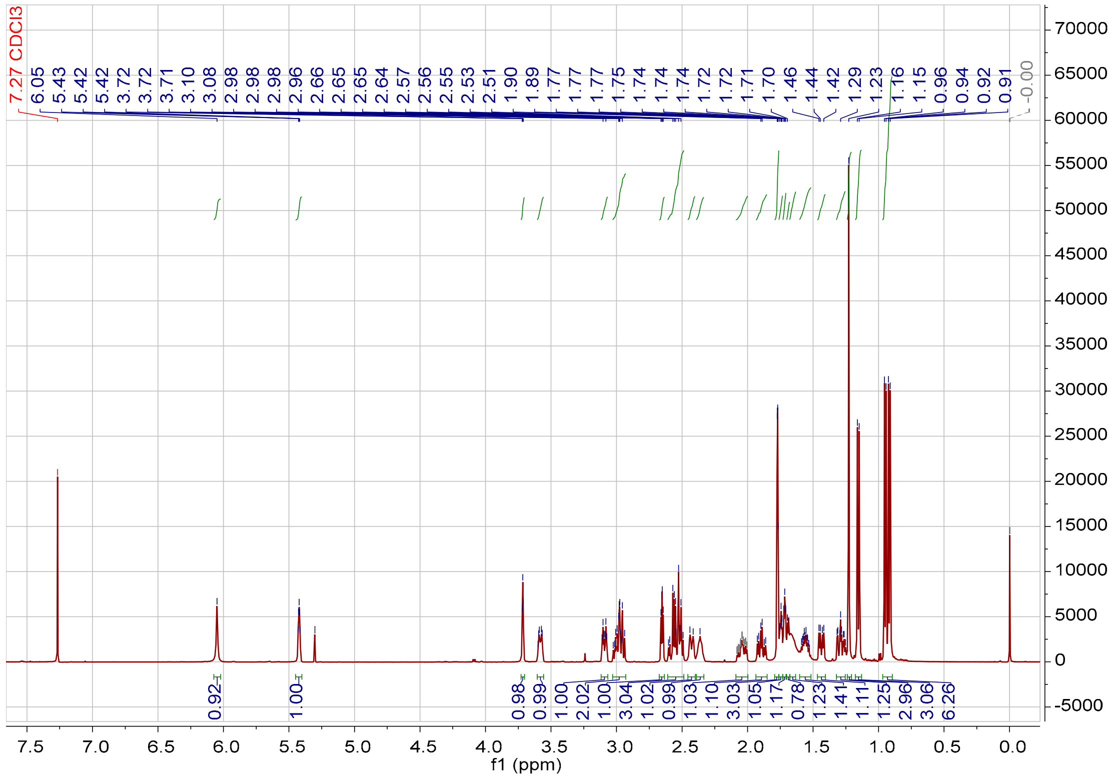

Figure S27. The ^{1}H-NMR spectrum of compound **4** in $CDCl_3$

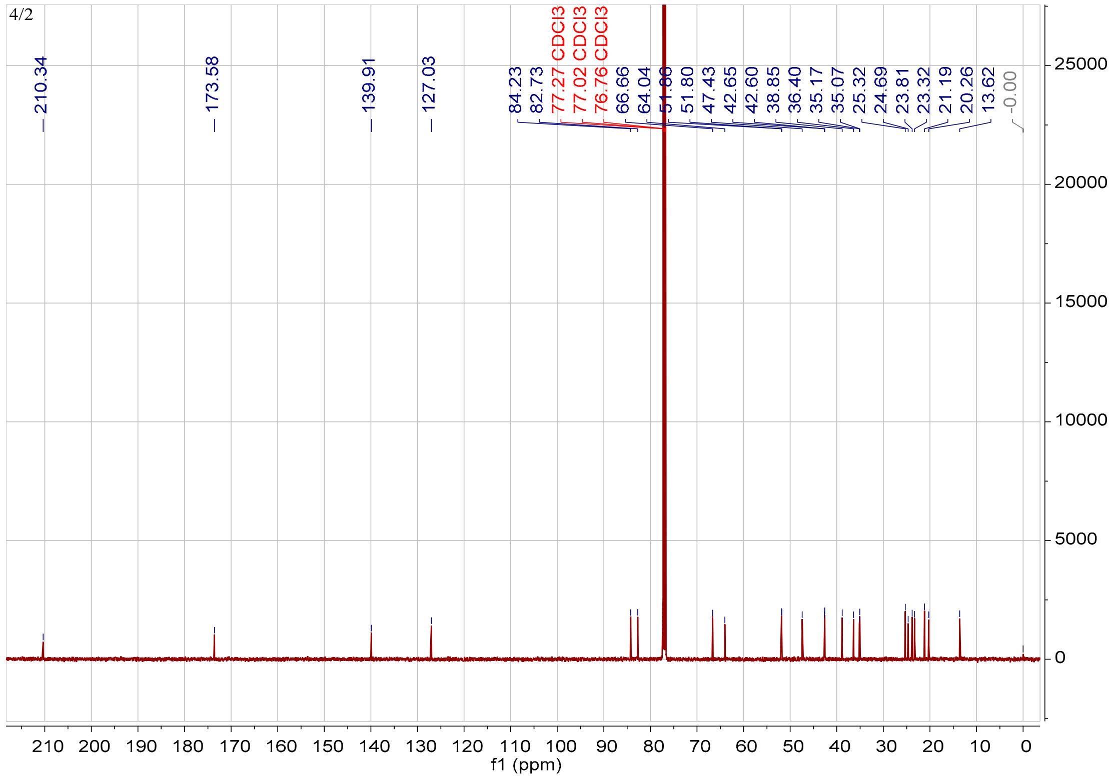

Figure S28. The ^{13}C-NMR spectrum of compound **4** in $CDCl_3$

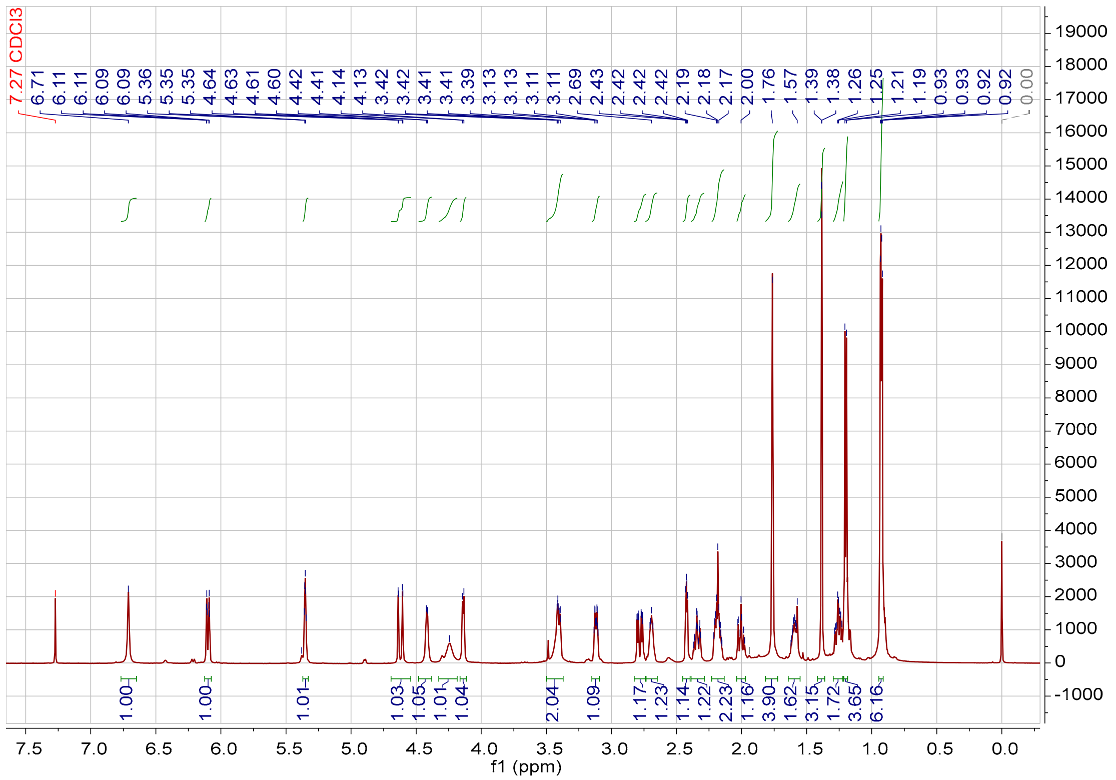

Figure S29. The ^{1}H-NMR spectrum of compound **5** in $CDCl_3$

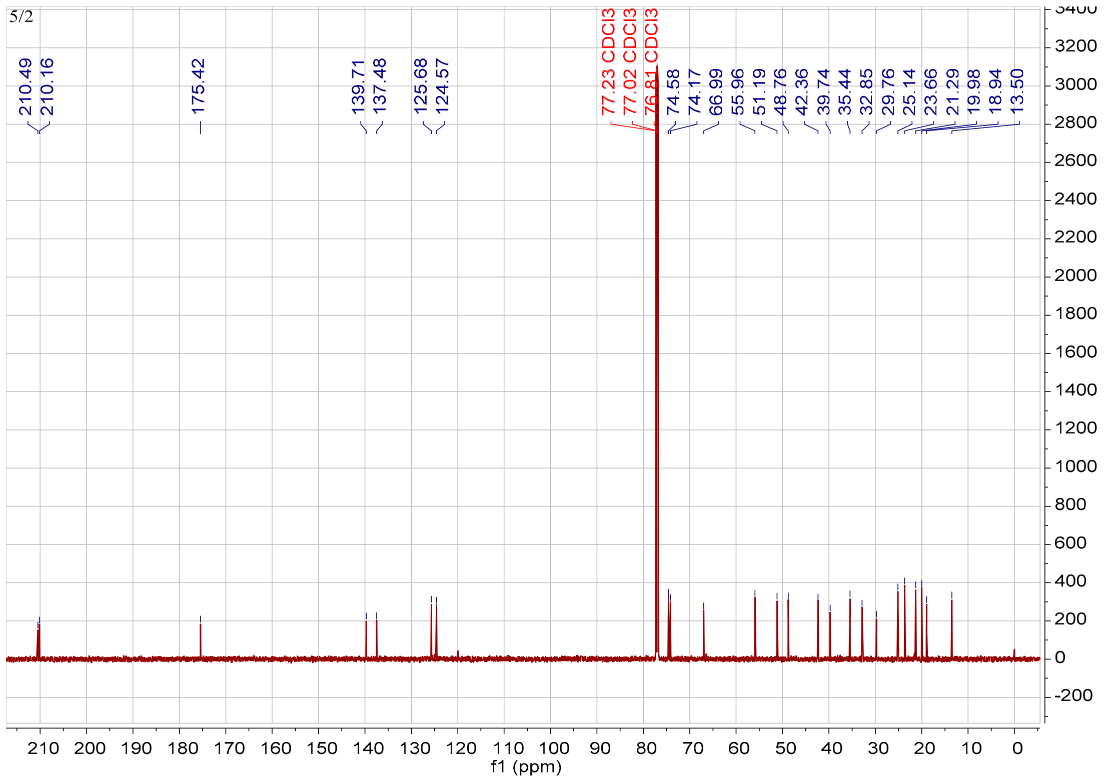

Figure S30. The ^{13}C-NMR spectrum of compound **5** in $CDCl_3$
